# Perspectives on the 2 × 2 Matrix: Solving Semantically Distinct Problems Based on a Shared Structure of Binary Contingencies

**DOI:** 10.3389/fpsyg.2020.567817

**Published:** 2021-02-09

**Authors:** Hansjörg Neth, Nico Gradwohl, Dirk Streeb, Daniel A. Keim, Wolfgang Gaissmaier

**Affiliations:** ^1^Social Psychology and Decision Sciences, Department of Psychology, University of Konstanz, Konstanz, Germany; ^2^Data Analysis and Visualization, Department of Computer Science, University of Konstanz, Konstanz, Germany

**Keywords:** 2x2 matrix, contingency table, framing effects, Bayesian reasoning, problem solving, scientific measurement, transparency, visualization

## Abstract

Cognition is both empowered and limited by representations. The matrix lens model explicates tasks that are based on frequency counts, conditional probabilities, and binary contingencies in a general fashion. Based on a structural analysis of such tasks, the model links several problems and semantic domains and provides a new perspective on representational accounts of cognition that recognizes representational isomorphs as opportunities, rather than as problems. The shared structural construct of a 2 × 2 matrix supports a set of generic tasks and semantic mappings that provide a unifying framework for understanding problems and defining scientific measures. Our model's key explanatory mechanism is the adoption of particular perspectives on a 2 × 2 matrix that categorizes the frequency counts of cases by some condition, treatment, risk, or outcome factor. By the selective steps of filtering, framing, and focusing on specific aspects, the measures used in various semantic domains negotiate distinct trade-offs between abstraction and specialization. As a consequence, the transparent communication of such measures must explicate the perspectives encapsulated in their derivation. To demonstrate the explanatory scope of our model, we use it to clarify theoretical debates on biases and facilitation effects in Bayesian reasoning and to integrate the scientific measures from various semantic domains within a unifying framework. A better understanding of problem structures, representational transparency, and the role of perspectives in the scientific process yields both theoretical insights and practical applications.

## 1. Introduction

Solving a problem simply means representing it so as to make the solution transparent.

(Simon, 1981, p. 153)

Human cognition is both empowered and limited by representations. Some of the greatest scientific discoveries—like the heliocentric cosmos, the Indo-Arabic number system, and the double-helix structure of the DNA molecule—involve fundamental changes in representations (Kuhn, 1962). Problems in logic and mathematics essentially ask for the explication of information that is provided in the problem formulation and are solved, or dissolved, by finding a superior problem representation (Polya, 1957). Although the history of psychology is littered with representational effects, the demands and rigidity of mental constructs are typically portrayed as a source of problems, rather than as opportunities for insight and solutions.

This article promotes a representational account for solving problems based on frequency counts and conditional probabilities that gravitates around the notion of a 2 × 2 matrix as its core construct. Just like the logical conditional (Wason and Johnson-Laird, 1972, p. 92), the humble 2 × 2 matrix is a chameleon that appears in many guises. Its structural simplicity is deceiving, as it accommodates an enormous manifold of measures and meanings. By explicating their shared structure, the model developed here integrates a wide variety of measures from different semantic domains in a unifying framework. As we will see, highly selective steps of filtering, framing, and focusing on particular parts of a 2 × 2 matrix eventually capture some scientific measure. Our model explicates this process and highlights the key role of adopting particular perspectives for gaining insights. Understanding how this mechanism simultaneously reveals and encapsulates some aspect of information that was implied by the original matrix builds conceptual bridges between domains and enables the transparent communication of scientific results. Before introducing our model, we recapitulate the role of representations in psychology and introduce a problem that we will revisit repeatedly throughout this article.

### 1.1. Reframing Representational Effects

The history of psychology is reflected in its representational constructs. Classic studies have lamented the rigidity of mental representations, and attributed their damaging effects to some lack of mental dexterity known as *Einstellung* (Luchins, 1942), *functional fixedness* (Duncker, 1945), or *negative transfer* (Bartlett, 1958). By contrast, desirable traits like creativity and productive thinking were seen as requiring a flexible reorganization of problem parts (Wertheimer, 1959). When the right representation is found, both chimpanzees and humans appear to stumble upon the problem's solution in a sudden flash of *insight* (Köhler, 1925).

Representations also provide the foundations for cognitive theories of thinking and problem solving. In the psychology of reasoning, people's responses to logical puzzles are based on a dynamic interplay of structure and content (Wason and Johnson-Laird, 1972). Beyond purely formal aspects of arguments, it has been shown that mental models of tasks and domains, the plausibility of premises, and concerns for relevance and linguistic pragmatics can both facilitate and inhibit logical thinking (Gentner and Stevens, 1983; Johnson-Laird, 1983; Sperber and Wilson, 1986; Nickerson, 1998). When specific contents increase the likelihood of valid conclusions, so-called facilitation effects were often attributed to the availability of particular representations (e.g., *pragmatic reasoning schemas*, Cheng and Holyoak, 1985), or to the evolution of domain-specific inference algorithms (e.g., a *cheater detection module*, Cosmides and Tooby, 1992).

Psychological investigations of judgment and decision making have been dominated by research on *heuristics and biases* (Tversky and Kahneman, 1974) and documented striking *framing effects* on decisions (Tversky and Kahneman, 1981). Early research on human problem solving was shaped by the *problem space hypothesis* (Newell and Simon, 1972), which postulates that we search and traverse a space of mental states until reaching our goal. Subsequent work addressed the benefits of diagrams (Larkin and Simon, 1987), contrasted the difficulty of representational isomorphs (Kotovsky et al., 1985), and studied tasks that distribute information across the mind and the external environment (Hutchins, 1995). Overall, researchers accumulated ample evidence for *representational effects* (Zhang and Norman, 1994): Different representations of a shared problem structure can cause dramatic differences in cognition and behavior.

**Figure 1:**
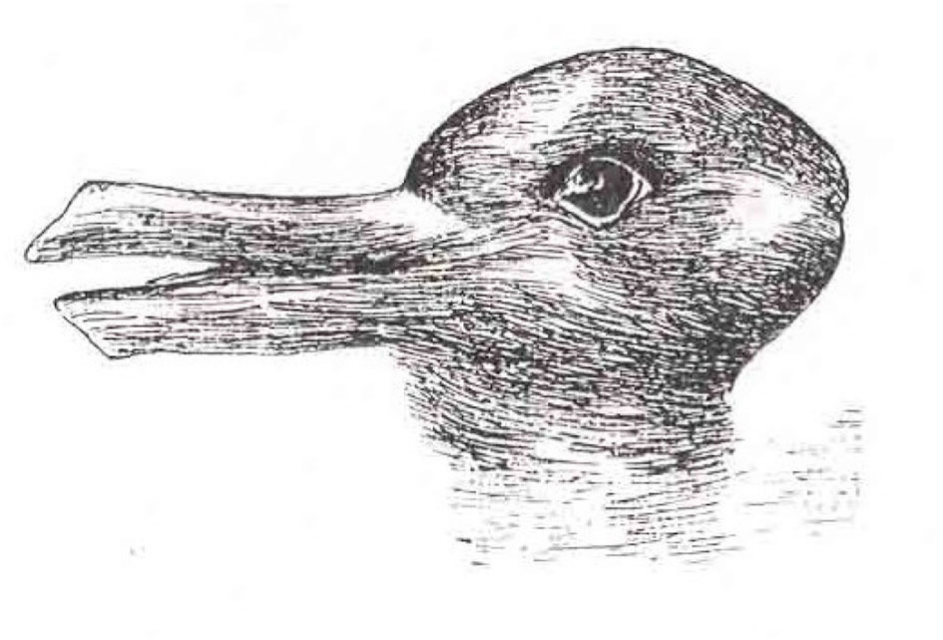
The rabbit-duck illusion (Jastrow, 1899).

A problem with representational accounts of cognition is that their explanations can be too narrow and specific. Although some explanation may be perfectly obvious, they remain hard to verbalize or generalize. When an ambiguous image can be viewed as either a rabbit or a duck (see Figure 1), a hint that the duck's beak can be seen as the rabbit's ears may ease the mental flip, but provides no material for scientific theories. Just as being too narrow is a problem, representational accounts that aspire to be general can easily get vacuous. For instance, when any possible conclusion can be explained as a valid deduction based on implicit premises (Henle, 1962) or in reference to “other things the speaker knows” (Braine and O'Brien, 1991, p. 192), overly wide and flexible explanations risk becoming circular (Smedslund, 1970). Similarly, most biases and fallacies can be explained as the result of improper representations or as resulting from deficient information processing (Fiedler and Juslin, 2006). Consequently, accounts that blur the boundaries between representational structures and processes are too permissive and vague to be useful. And although Simon (1981) rightly insists that problems are solved by making their solution transparent, it is far from simple to explicate a problem's mental representation, let alone its transparent solution.

How can we capitalize on Simon's insight that transparent representations are solving problems? In this article, we essentially promote a notion of positive framing effects. In our view, a productive representational account requires a revolution, in the literal sense that implies a reversal or shift in perspective. Rather than gravitating around a particular problem and examining its possible representations, we must anchor our investigations in the analysis of shared representational structures. Shifting from focusing primarily on tasks to pivoting around particular representations has immediate benefits: Starting from the representation avoids getting trapped in problem-specific trivialities and allows for non-circular accounts of representational transparency. Instead of serving as convenient *post-hoc* explanations for observed behavior, representational constructs can be studied independently and prior to specific tasks. Ideally, this will illuminate aspects that were obscured before and replace retrospective explanations by genuine predictions. And rather than portraying representational isomorphs as problems to-be-solved, the discovery of a common underlying structure provides opportunities for clarifications and builds conceptual bridges between semantic variants of tasks and domains.

To illustrate this approach, this article proposes an abstract model for analyzing problems that rely on binary frequency counts and probabilistic measures derived from them. Our model is anchored in the representational construct of a 2 × 2 matrix, which we employ to reframe a variety of measures and problems. As this construct is shared across many semantic domains, explicating its structural features and the mechanisms operating upon them illuminates and links many concepts and tasks that are typically treated in isolation. Before we can unfold this model, we introduce a problem that allows illustrating the steps and tasks involved in our approach. But rather than merely serving as a sandbox, this problem has provoked intense theoretical debates within psychology and beyond, and will be rendered more transparent by our framework.

### 1.2. The Mammography Problem

The *mammography problem*(Eddy, 1982) is the drosophila of a research tradition that has been haunting both psychology and clinical diagnostics for decades. Typical problems in this tradition ask for inferring the probability of a potential cause (e.g., some medical condition *C*) given an observed effect (e.g., a positive test result *T*). In its standard form, the problem provides a condition's *base rate* (e.g., the *prevalence* of cancer, *p*(*C*) = 1%), the conditional probability of correctly detecting the condition's presence (e.g., the mammography test's *sensitivity*, *p*(*T*|*C*) = 80%), and the conditional probability of falsely detecting the condition in its absence (e.g., the test's *false positive rate*, *p*(*T*|¬*C*) = 9.6%). Solving the problem consists in computing the value of the conditional probability *p*(*C*|*T*), which denotes the test's *positive predictive value* (PPV). Such problems are often framed as requiring “Bayesian reasoning,” as their mathematical solution can be derived by *Bayes' theorem*:

$$\begin{array}{l} p\left(C|T\right)=\frac{p\left(C\right)\cdot p\left(T|C\right)}{p\left(C\right)\cdot p\left(T|C\right)\;+p\left(\neg C\right)\cdot p\left(T|\neg C\right)}\\ \;=\frac{0.01\cdot 0.80}{0.01\cdot 0.80\;+\;\left(1-0.01\right)\cdot 0.096}\approx \;7.8\%. \end{array}$$

In a seminal paper, Gigerenzer and Hoffrage (1995) devised 15 variants of this problem and presented them in different formats (see Table 1). Importantly, they reported facilitation effects for two types of representational changes: Both expressing the problem in *frequency formats* (or *natural frequencies*) and using a short version containing fewer numbers (aka. *short menu*) boosts the rate of correct solutions (see the meta-analysis by McDowell and Jacobs, 2017). Whereas, Gigerenzer and Hoffrage (1995) describe their manipulations in terms of information representation, they explain the observed effects primarily as computational facilitation. For instance, the algorithm for solving the problem in frequency formats simplifies to:

$$\begin{array}{l} p\left(C|T\right)=\frac{n\left(T\cap C\right)}{n\left(T\right)}=\frac{n\left(T\cap C\right)}{n\left(T\cap C\right)\;+\;n\left(T\cap \neg C\right)}\\ \;=\frac{8}{8\;+\;95}=\frac{8}{103}\approx \;7.8\%. \end{array}$$

The *mammography problem*'s notoriety has many reasons. For both experimental participants and medical professionals, the problem seems of high practical relevance, but is frustratingly difficult. Most naïve respondents estimate its solution to be around 70 or 80%, thus misjudging the true value by an order of magnitude. Theoretically, the error committed in the context of such problems has been described by a confusing array of concepts—including *base rate neglect* (Kahneman and Tversky, 1973), *base rate fallacy* (Bar-Hillel, 1980), and *insensitivity to prior probability* (Tversky and Kahneman, 1981)—and attributed to an *inverse fallacy* (Eddy, 1982) or a heuristic of *representativeness* (Kahneman and Tversky, 1972b). Even when the problem's solution is known, the discrepancy between the mammography's high sensitivity and its low PPV remains perplexing. In addition to the theoretical challenge of explaining people's poor performance, researchers in applied psychology, clinical diagnostics, and information visualization face the practical challenge of improving it. In numerous attempts to train people (e.g., Sedlmeier and Gigerenzer, 2001; Ruscio, 2003; Sirota et al., 2015) or support their performance by visual aids (e.g., Brase, 2008; Moro et al., 2011; Garcia-Retamero and Hoffrage, 2013; Binder et al., 2015, 2020; Böcherer-Linder and Eichler, 2017; Eichler et al., 2020), solutions rates remained frustratingly low (e.g., Micallef et al., 2012; Khan et al., 2015; Weber et al., 2018). Thus, despite considerable progress, it is still controversial to what extent humans are able to solve such problems, how they perform the required calculations, and which aspects of the task, person, or task environment help or hinder their performance (see Navarrete and Mandel, 2016; McDowell and Jacobs, 2017, for reviews).

**Table 1:** Three versions of the *mammography problem* (from Gigerenzer and Hoffrage, 1995, Table 1, p. 688), and an overview of the information provided and required for solving each version (probabilities *p* in blue, frequencies *n* in green, and parts of required solutions in red).

		**Problem description**	**Information**	% correct^*^
Standard Probabilities	(a) (b) (c)	The probability of breast cancer is 1% for a woman at age forty who participates in routine screening.? If a woman has breast cancer, the probability is 80% that she will get a positive mammography. If a woman does not have breast cancer, the probability is 9.6% that she will also get a positive mammography. A woman in this age group had a positive mammography in a routine screening. What is the probability that she actually has breast cancer? ____%	*p*(*C*)=0.010 *p*(*T*|*C*)=0.800 *p*(*T*|¬*C*)=0.096 *p*(*C*|*T*)=?	4%
Natural Frequencies	(a) (b) (c)	10 out of every 1,000 women at age forty who participate in routine screening have cancer. 8 of every 10 women with breast cancer will get a positive mammography. 95 out of every 990 women without breast cancer will also get a positive mammography. Here is a new representative sample of women at age forty who got a positive mammography in routine screening. How many of these women do you expect to actually have breast cancer? ____out of ____%	*n*(*C*)=10 *N*=1,000 *n*(*C* ∩ *T*)=8 *n*(*C*)=10 *n*(¬*C* ∩ *T*)=95 *n*(¬*C*)=990 *n*(*T* ∩ *C*)=? *n*(*T*)=?	24%
Short Frequencies	(d) (e)	103 out of every 1,000 women at age forty get a positive mammography in routine screening. 8 of every 1,000 women at age forty who participate in routine screening have breast cancer *and* a positive mammography. Here is a new representative sample of women at age forty who got a positive mammography in routine screening. How many of these women do you expect to actually have breast cancer? ____ out of ____%	*n*(*T*)=103 *N*=1,000 *n*(*C* ∩ *T*)=8 *N*=1,000 *n*(*T* ∩ *C*)=? *n*(*T*)=?	36%

* *Estimates of correct answer rates (from McDowell and Jacobs, 2017) for problems in this format*.

We contribute to these debates by proposing new perspectives on the problem. Rather than focusing on differences between representational formats, we explicate the steps and processes that lead from the provided information (i.e., probabilities or frequencies) to the measures required for solving the problem. As we will show, this illuminates the geometric nature of the underlying problem representation in ways that explain both the problem's difficulty and the observed facilitation effects. As a collateral benefit, our analysis can be applied to related problems and allows defining a large variety of scientific measures from seemingly distinct domains in a unified framework. Our account is embedded in a broader model that emphasizes the role of 2 × 2 matrices as a key construct of scientific inquiry.

## 2. The Matrix Lens Model

This article introduces an abstract *matrix lens model* of scientific inquiry. As an analytic device, this model explicates the steps and processes that we perform when solving problems based on frequency counts, binary contingencies, and probabilistic measures derived from them. The core representational component of our model is the structural construct of a 2 × 2 matrix that frames and sculpts a large variety of measures in seemingly distinct tasks and domains. The key mechanism invoked by our framework is the adoption of particular perspectives on parts of this matrix. By selectively focusing on some aspects while ignoring others, highly specialized measures trade-off gains in depth and resolution with losses in context and scope. As a consequence, the transparent communication of such measures must explicate the perspectives encapsulated in their derivation.

**Figure 2:**
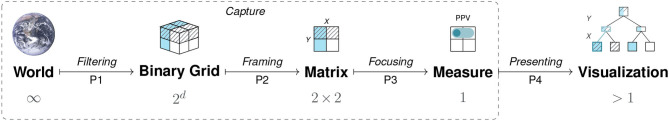
The *matrix lens model* describes scientific inquiries that reduce complexity in several steps by adopting increasingly specific perspectives on particular aspects of the world. Its initial steps reduce the dimensions of explicitly represented information by *filtering, framing*, and *focusing* (P1–P3) to *capture* a particular measure (e.g., a diagnostic test's *positive predictive value*, PPV). By contrast, the final step of *presenting* (P4) can widen the scope by creating representations that are *transparent* when explicating the perspectives adopted during the measure's derivation.

Figure 2 illustrates the steps of our model as a pipeline of adopting increasingly specific perspectives. When providing a numeric answer to a scientific question, we dramatically reduce the world's complexity by selecting and zooming into relevant aspects to eventually capture the value of some measure (e.g., PPV). An initial step of *filtering* (P1) categorizes some population of elements on binary dimensions to yield a binary grid of frequency counts as a prerequisite for the model's two main steps, whose geometric nature corresponds to the visual process of adopting particular perspectives. A second *framing* step (P2) selects and arranges dimensions to construct a specific 2 × 2 matrix. Given this matrix, a *focusing* step (P3) further selects and highlights some particular aspect to derive a quantitative measure. The value of this measure implicitly contains the entire chain of transformations and thus encapsulates the perspectives adopted in the measure's derivation. An additional step of *presenting* (P4) communicates the measure as a scientific result. Whereas, the model's three initial steps (P1–P3) reduce complexity—by selectively carving out, organizing and compressing information—its final step (P4) widens the scope by adding information and providing an interpretation. As a prescriptive consequence, a measure's verbal or visual presentation is *transparent* when explicating the perspectives that were encapsulated in its derivation.

Capturing some noteworthy aspect of the world by viewing it through the lens of a 2 × 2 matrix requires a mix of numeric and representational skills. Selecting the right measure out of a large variety of options typically requires both task-related experience and domain-specific knowledge. Although the measures deemed relevant and their labels vary between tasks and domains, the basic steps and mechanisms mostly remain the same. In the following, we first illuminate the structural elements of each step by abstracting from the content and semantics of specific tasks. This will portray the act of scientific measurement as a deliberate, strategic, and intricately coordinated process that encompasses different levels, decisions, and parameters. Just like a photographer is not merely pointing a lens in the direction of an object of interest and then randomly triggers the shutter, a scientist aiming to answer a question is not randomly screening data and computing metrics that may or may not answer a question. In practice, and particularly in experts, this process may nevertheless unfold in an automatic and intuitive fashion. This allows for glitches and errors, if something breaks down or is led astray, as well as for systematic biases, due to schematic processes and preferred perspectives. Overall, our model emphasizes the selective and directional elements of scientific investigations and reveals scientific insights as a matter of adopting and presenting particular perspectives.

### 2.1. Filtering

The reductionist nature of our model is most obvious in its initial step of *filtering*, which categorizes a population of elements on binary dimensions and acts as a sieve for all subsequent steps. The object being filtered is defined as some *population* of elements that can be measured on our dimensions of interest. Although this population can comprise any well-defined set of elements, we usually encounter subsets of *samples* and *elements* that represent events or individuals. Measuring elements requires a *dimension* of interest and a *scale* that assigns values to elements. An elementary type of measurement is *categorization*, which uses some rule to assign or arrange elements into groups.

**Figure 3:**
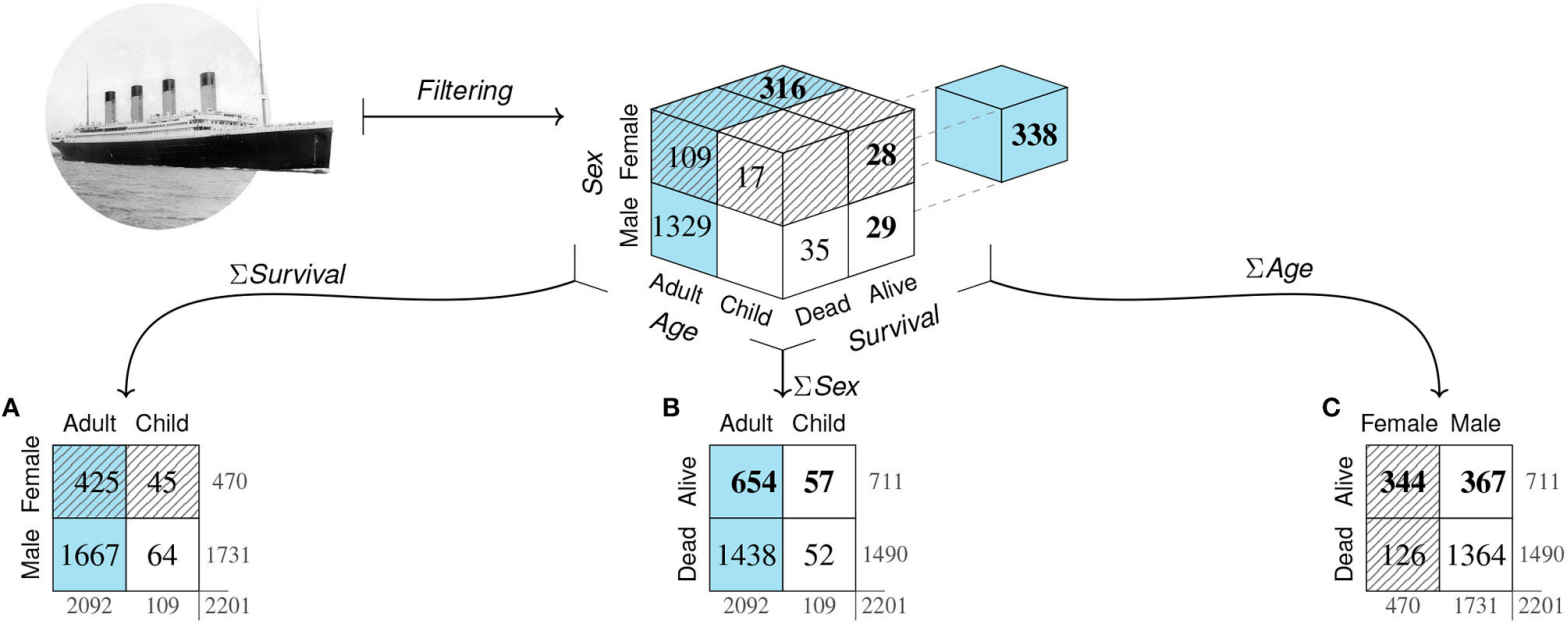
*Filtering* the population of *N* = 2, 201 passengers of the RMS *Titanic* on *d* = 3 binary dimensions and *framing* the resulting frequency grid as three distinct 2 × 2 matrices. The top cube shows the frequency counts of eight subgroups resulting from categorizing all elements by the binary variables *Age, Sex*, and *Survival*. Due to aggregation, all arrows are uni-directional. Arrows from cube to matrices show the three possible two-dimensional projections along each of the cube's axes. The three 2 × 2 matrices **(A–C)** result from adding the frequency counts of the collapsed dimension. (Color marks *Adult* category; pattern marks *Female* category; bold font marks *Alive* category. Titanic image adapted from: https://commons.wikimedia.org/wiki/File:RMS_Titanic_3.jpg).

The elements of a population can be categorized in many different ways. In this paper, we limit ourselves to cases of *binary* categorization in which the categories employed are dichotomous, exhaustive, and mutually exclusive, so that each element falls into exactly one of two categories on any dimension of interest. As an example, suppose we aimed to investigate what may have contributed to surviving the sinking of the RMS *Titanic* in 1912. Our population of elements consists of the *N* = 2, 201 passengers on board of the *Titanic* on its fatal maiden voyage. Suitable dimensions of interest could be the age, sex, or class of each passenger (see Dawson, 1995). To satisfy the constraint of binary dimensions, any variable describing the passengers must be dichotomous. Although the variable *Age* is continuous when expressed in terms of years, it can be categorized into *Adult* vs. *Child*. Similarly, the variable *Sex* is often categorized into *Female* vs. *Male*, despite allowing for finer distinctions. A key outcome variable in this example is each passenger's *Survival*, categorized into *Alive* vs. *Dead*. Cross-classifying all elements on *d* binary dimensions arranges them in a *d*-dimensional grid. The top cube of Figure 3 illustrates this for *d* = 3 dimensions. As each of three variables contains two categories and all of their 2^*d*^ = 8 possible combinations exist, the population is dissected into eight sub-cubes that show the frequency counts of individuals for every category combination. Interestingly, any two-dimensional visualization of a three-dimensional problem introduces artifacts that are based on properties of the representation, rather than the problem. For instance, depicting categories as the cells of a cube implies an element of spatial clustering that mere classification does not provide. Similarly, an issue of arranging categories arises due to constraints of viewing a 3*d*-object from a particular perspective. Here, the sub-cube in the hidden lower corner of the population cube—which is obscured by the currently adopted angle of view and thus drawn separately, shifted to the right—shows that 338 male adults survived the disaster. The tension between the properties of a represented object and the effect of highlighting or occluding some aspects by choosing a particular representation forms a recurring theme throughout this article: Whereas, some subjective elements—like choosing particular dimensions or binary cut-off values—are an inevitable consequence of reducing a multi-faceted world to a 2^*d*^-grid, merely representational constraints often occur as side-effects and can be mitigated by choosing other representations.

Overall, the initial step of *filtering* imposes a binary perspective upon the world. Although the range of questions that can be addressed within this framework remains substantial, it is clear that this step is highly selective and reduces complexity by many orders of magnitude. By rendering chosen variables from shades of gray as either black or white, certain aspects of the world are emphasized while others are ignored. For instance, if the variable of a passenger's *Class* is available but not considered in this step, it is lost and cannot be recovered later.

### 2.2. Framing

A second step of *framing* reduces our object of inquiry to two dimensions by transforming the binary grid into a 2 × 2 matrix. When the elements of our population are clustered as a three-dimensional cube, adopting perspectives on this cube corresponds to viewing it from particular directions. Figure 3 illustrates this step geometrically as projections along each of the cube's dimensions. Crucially, each of the three resulting 2 × 2 matrices (Figures 3A–C) is an abstraction of the categorical information that achieves simplification by further aggregating over one of the cube's dimensions. As the three projections are orthogonal, any two 2 × 2 matrices provide the marginal sums of the third matrix, but do not allow reconstructing it without additional information. Again, our *Titanic* example illustrates that adopting particular perspectives on an object implies both reduction and specialization. Switching to a different representation can sacrifice, hide, or reveal information that was implicit before. Additionally, changing representations imposes new constraints that can illuminate or obscure particular aspects, but may also introduce representational artifacts. As we shall see, each 2 × 2 matrix allows answering a wide range of questions. But all insights provided by increasingly detailed comparisons and metrics come at the price that other aspects are obscured or lost. Thus, the benefits of adopting any particular perspective incur potential costs of neglecting or abandoning alternative view-points and interpretations.

When categorizing the elements of a population on two binary dimensions, their cross-tabulation as a *2* × *2 matrix* provides “the crudest possible division” (Pearson, 1904, p. 21) into four sub-groups, with each table cell displaying the frequency count of the corresponding category combination. The core construct of our model is also known as a binary *contingency table* (e.g., Everitt, 1977; Powers, 2011)—a term coined by Karl Pearson, who pioneered its statistical analysis (in Pearson, 1904). Alternatively, the same four-fold table is also known as *confusion matrix* (e.g., Fawcett, 2006; Ting, 2011; Chicco, 2017) or *error matrix* (e.g., Stehman, 1997). To anyone familiar with the literature on the subject, these latter terms seem uncannily appropriate, as they not only apply to the table itself, but also characterize the plethora of measures and interpretations it subsequently spawned, and even provide an apt description of the state of mind of many of its students. We see three types of reasons for the confusing nature of 2 × 2 matrices:

1. *Structural reasons*: A first source of errors is the deceptive simplicity of its structure. While any 2 × 2 matrix provides a “simple four-fold division of the universe” (Pearson, 1904, p. 3), actually *framing* this construct implies (a) the selection of two binary dimensions, and (b) their arrangement in a spatial layout. As there exists no standard layout of a given 2 × 2 matrix, swapping the order of its dimensions and their categories allows for 2^3^ = 8 different ways of representing the same information (see Supplementary Figure 1). Although all these spatial variants are mirror images or rotations of a single 2 × 2 matrix, this flexibility in expression allows for a multiplicity of surface structures that differ between authors, applications, and domains.
2. *Semantic reasons*: A second source of confusion is that seemingly similar surface structures vary substantially in their semantic interpretations. Both the specific dimensions mapped to the axes of a 2 × 2 matrix and the relations between their categories influence its meaning. For instance, many binary distinctions (e.g., *Alive/Dead, Adult/Child*) imply preferences that carry over to the perception of corresponding matrix cells. Similarly, particular combinations of categories (e.g., *Adult/Alive* vs. *Child/Dead*) give rise to further evaluations. Thus, the four cells of an interpreted matrix can vary both categorically (e.g., positive/negative, correct/incorrect, etc.) and as matters of degree (e.g., some cells are more relevant than others). Within our visual metaphor, we can think of these semantic aspects as re-introducing colors, patterns, or shades to a 2 × 2 matrix, and exuding substantial implications beyond its binary structure.
3. *Terminological reasons*: A third and particularly vexing type of reasons for the confusing nature of 2 × 2 matrices is that different semantic domains not only frame different matrices, but also label the resulting measures by distinct concepts. As a consequence, the same measures often appear in different terminological disguises, rendering their identification and selection difficult and error-prone.

Fortunately, these structural, semantic, and terminological sources of confusion can be reduced by adopting an analytic and functional perspective on a shared representational construct. In the following sections, we use a framed 2 × 2 matrix as a foundation for tackling each of the confusions in turn. From a functional viewpoint, we can ask: Which generic goals or tasks are supported by a 2 × 2 matrix? Regarding semantic issues, we will explicate the typical mappings and terminologies of different domains. Before addressing the semantic and terminological issues (in sections 3, 4), the next step of *focusing* provides the key mechanism of our model.

### 2.3. Focusing

Given a well-defined 2 × 2 matrix, *focusing* on parts of this structure supports distinct tasks that reveal increasingly specific aspects. These tasks remain implicit when using mathematical concepts and formulas to define measures based on the contents of matrix cells. By contrast, our model explicates these tasks and shows how the measures arise by adopting particular perspectives on the 2 × 2 matrix. Whereas, a numeric value encapsulates the perspective adopted in its derivation, our structural approach illuminates both the specific detail provided by each measure and its limits due to ignoring all other aspects.

**Figure 4:**
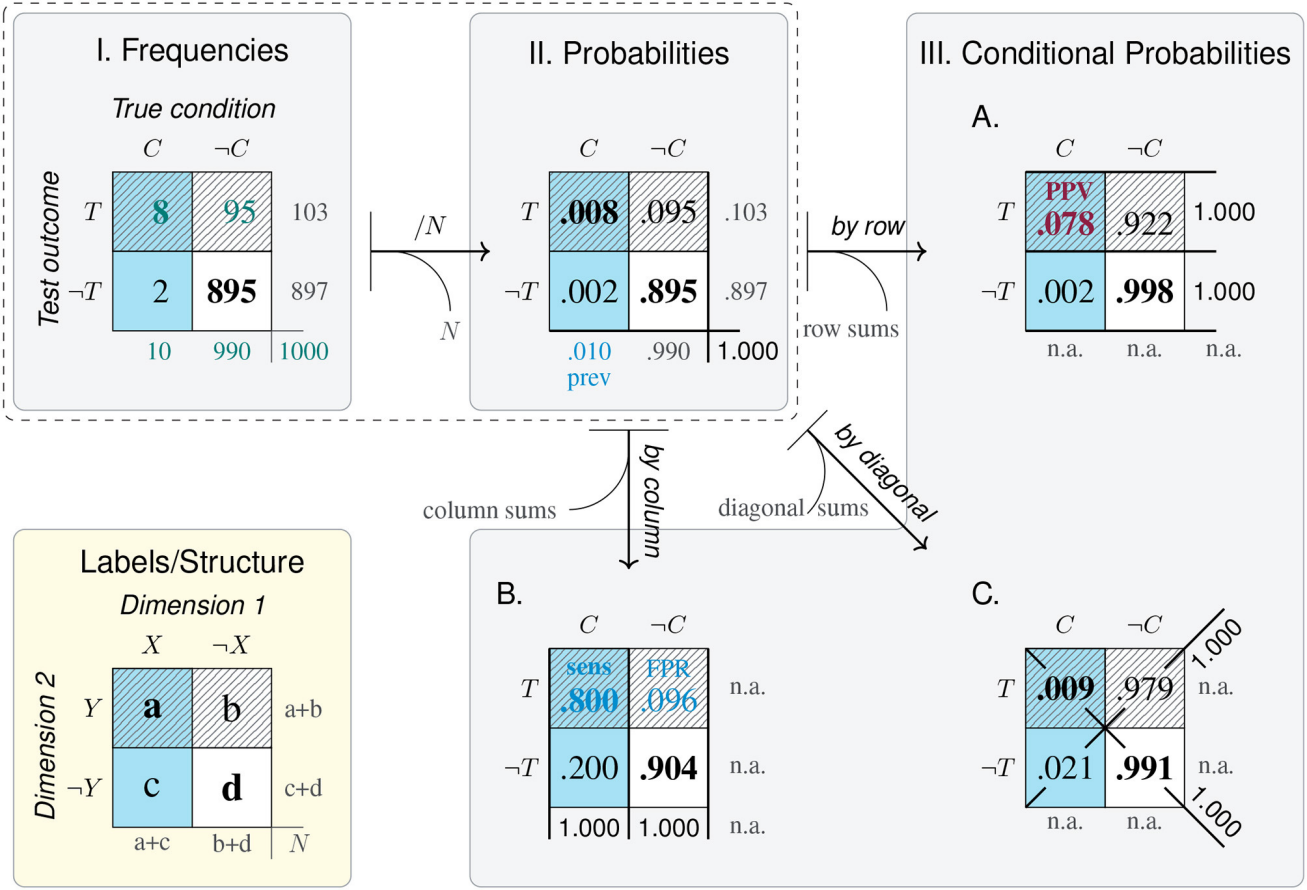
The structure of the 2 × 2 matrix and labels for its dimensions, categories, and cells. Numbered panels express the *mammography problem* in a 2 × 2 matrix framework to illustrate the transformations of cell values from frequency counts **(I)**, to probabilities **(II)**, and conditional probabilities **(III)**. Arrows represent the direction of adopted perspectives and numeric transformations, with curved exits indicating information that is lost by a transformation and needs to be added when moving in the opposite direction. Cell background color marks category *C* (cancer present); pattern marks category *T* (positive test outcome); bold font marks category correspondence (correct cases). Numbers shown in blue, green, and red mark the provided probabilities, corresponding frequencies, and the solution of the problem, respectively.

Before explicating the *mammography problem* in our model, we introduce some abstract nomenclature. The highlighted panel of Figure 4 provides abstract labels for the dimensions, categories, and cells of a 2 × 2 matrix. In the absence of any semantic interpretation, the lowercase letters a, b, c, and d describe a 2 × 2 matrix by denoting the frequency counts of its top-left, top-right, bottom-left, and bottom-right cell, respectively. Using a matrix-based framework for structuring our analysis primarily provides us with a methodological tool. Thus, rather than claiming that the 2 × 2 matrix provides a superior type of visualization (see e.g., Binder et al., 2020; Eichler et al., 2020, for comparisons between alternatives), we use its geometric potential for distinguishing between locations and directions.

As a result of framing, we can refer to the dimensions and categories of a 2 × 2 matrix by combining the corresponding labels. Figure 4I cross-tabulates the primary dimension of a *True condition* (consisting in the presence or absence of *cancer*, *C* vs. ¬*C*) with a secondary dimension of a positive or negative *Test outcome* (*T* vs. ¬*T*) to yield a 2 × 2 matrix containing the four possible combinations of all category levels. Thus, the cell label ‘a' and the number of elements in set *C* ∩ *T* are two ways of referring to the same frequency count. The numeric values in Figure 4I result from reconstructing the *mammography problem*'s probability information in terms of frequencies. When assuming a sample of *N* = 1, 000 women of the target population, a cancer prevalence of *P*(*C*) = 1% implies that 10 of them are expected to have cancer [*N*·*P*(*C*) = 1, 000·0.01 = 10]. Next, the sensitivity of the screening test *p*(*T*|*C*) = 0.80 suggests that a = 10·0.80 = 8 of the women with cancer also test positively (*C* ∩ *T*). For the *N*−10 = 990 women without cancer, the probability for a positive test is *p*(*T*|¬*C*) = 0.096, so that b = 990·0.096 ≈ 95 receive a false positive test result (¬*C* ∩ *T*). All other frequencies of the 2 × 2 matrix can then be computed, as the four elementary cells add up to the total number of individuals in the population (i.e., *N* = a + b + c + d = 1, 000 women), as do the sums of its row and column margins (e.g., *N* = 103 positive + 897 negative test outcomes). Thus, Figure 4I provides a well-defined 2 × 2 matrix that estimates the frequency counts of the *mammography problem* for a sample of *N* = 1, 000 women.

Which types of tasks are supported by a 2 × 2 matrix? And which numeric transformations are required to address these tasks? The panels of Figure 4 identify five types of tasks in a generic fashion:

1. *Frequencies*: The only type of task directly supported by a 2 × 2 matrix is the evaluation of frequencies. For instance, Figure 4I shows that—given a population of *N* = 1, 000 women—a majority of *d* = 895 of them do not have cancer and receive a correct negative test result (¬*C* ∩ ¬*T*). Adding cells of joint frequencies across rows or columns allows comparing frequency counts between category levels. For instance, the marginal sums reflect that there are fewer women with than without cancer (10 vs. 990), and fewer with a positive than with a negative test result (103 vs. 897).
2. *Proportions and probabilities*: A second type of task supported by the 2 × 2 matrix is the assessment and comparison of proportions. Expressing frequencies in terms of proportions facilitates comparisons of relative magnitudes by standardizing cell values and their sums to a reference value. As the frequency counts of the four original cell values add up to the population size *N*, dividing them by *N* normalizes their values to a sum of 1, allowing for their interpretation as the probability of each category combination (see Figure 4II). As this transformation leaves all relative proportions within the 2 × 2 matrix intact, all row and column values still add up to their marginal sums. Some of these marginal sums convey interesting facts about the original 2 × 2 matrix. For instance, adding the probabilities of the left column yields the *prevalence* of cancer in the population [*P*(*C*) = 1%], and adding those of the top row reflects the test's *bias* for positive outcomes [*P*(*T*) = 10.3%]. However, the benefits of convenient expression and comparison of cell values come at the cost that all information regarding the population size *N* is lost in the transformation.
3. *Correspondence*: The tabular structure of the 2 × 2 matrix primarily suggests combining rows or columns of cell values, but combining other configurations is often informative. A special type of aggregation consists in adding the *diagonals* of a 2 × 2 matrix (i.e., the frequencies a + d vs. b + c in Figure 4I, or their corresponding proportions in Figure 4II). In the *mammography problem*, the diagonals mark the *correspondence* between a woman's true condition and her test outcome. Any instance in the top-left or bottom-right cells (i.e., the counts of a and d) represents a woman with a *correct* test result (due to the correspondence *C* ∩ *T* or ¬*C* ∩ ¬*T*), while any element in the top-right or bottom-left cells (i.e., b and c) represents a woman with an *incorrect* test result (due to a lack of correspondence, ¬*C* ∩ *T* or *C* ∩ ¬*T*). Whereas, *correctness* is a categorical property of each individual (Rescher, 1998), accumulating the groups of all correctly diagnosed women (a + d = 903) and all incorrectly diagnosed women (b + c = 97), and computing their proportion (by dividing both sums by *N*), yields continuous measures of *accuracy* (90.3%) and *error rate* (9.7%). Both measures fit into our increasingly familiar pattern of gaining abstraction while sacrificing detail: On one hand, they provide easily interpretable values on a convenient scale from 0 to 1. On the other hand, the normalization and aggregation in their derivation obscure not just the population size *N*, but all differences between accurate instances (a vs. d) or inaccurate instances (b vs. c) have also vanished.
4. *Conditional probabilities*: A key transformation of a 2 × 2 matrix consists in dividing its cell values by its marginal sums to obtain *conditional probabilities* (see Figure 4III). The three sub-panels A–C differ in the reference class on which the cell values (of Figures 4I,II) were conditionalized. Adopting a *by row, by column*, or *by diagonal* perspective on a 2 × 2 matrix normalizes its values in the corresponding direction (i.e., the rows, columns, or diagonals of Panels A, B, and C, add to a sum of 1).
  As we explicate the semantics of diagnostic measures and other domains later (in sections 3, 4), we only contrast two conditional probabilities that matter in the context of the *mammography problem* here. By adopting a *by column* perspective on the 2 × 2 matrix, Panel B normalizes cell values on the presence or absence of cancer (*C* vs. ¬*C*). Thus, the top-left cell of Panel B shows that the conditional probability of receiving a positive test result given that a woman has cancer is *P*(*T*|*C*) = 80.0%. This is the *sensitivity* of the mammography test provided by the original problem formulation (in blue). By contrast, Panel A adopts a *by row* perspective and normalizes its values on the possible outcomes of a mammography test (*T* vs. ¬*T*). Thus, the top-left cell of Panel A shows that the conditional probability of having cancer given a positive test result is *P*(*C*|*T*) = 7.8% (in red). This is the test's *positive predicted value* (PPV) and the solution to the original problem.
  As with previous transformations, computing probabilities that normalize values by a particular perspective yields highly specialized measures that render comparisons in one direction simple and transparent, but drop any information regarding the base rates of rows, columns, and diagonals. For instance, whereas Figures 4I,II show that women with cancer (*C*) and with a positive test result (*T*) are clear minorities, this information is lost in the transformations to Figure 4III.
5. *Contingencies*: Detecting the degree of *covariation* or *contingency* between events is an important adaptive task. In the context of a 2 × 2 matrix, detecting contingency concerns the relation between its dimensions. In the absence of contingency, both dimensions are independent of each other, whereas the presence of contingency implies a dependency, association, or correlation between them. Contingency-related questions are answered by assessing differences in conditional probabilities (e.g., by subtracting or dividing two conditional probabilities) or computing more comprehensive metrics (e.g., the χ^2^-score, or the *Matthews correlation coefficient*, MCC). We discuss some of these metrics in the context of classification and diagnostics (in section 4.1).
  Importantly, any measure based solely on the values of a transformed 2 × 2 matrix inherits both the benefits and limitations of its origin. Hence, any measure based exclusively on the conditional probabilities of Panel A may be highly informative for answering questions that are *conditionalized* on a specific *Test outcome*, but is useless or misleading for addressing tasks that require the absolute frequency or proportion of women with vs. without cancer or with vs. without a particular test outcome.

The five types of tasks enabled by a 2 × 2 matrix reach from relatively simple comparisons (based on the frequency or probability of cells or cell combinations) to more complex judgments (involving assessments of correspondence, conditional probability, and contingency). However, solving a specific problem does typically not recruit all of these tasks. For instance, solving the *mammography problem* primarily requires adopting a particular perspective on a 2 × 2 matrix that cross-classifies the target population's health condition *C* by test outcomes *T*. Comparing the values provided and required in Figures 4II,III reveals the essence of the *mammography problem*: The test's sensitivity for detecting cancer *p*(*T*|*C*) is conditionalized on a low cancer prevalence *P*(*C*), whereas the required PPV *p*(*C*|*T*) is conditionalized on a proportion of positive test results *P*(*T*) that is more than ten times higher than the prevalence. More generally, a conditional probability *p*(*C*|*T*) typically differs (a) from the unconditional probability *P*(*C*)—unless *C* and *T* are independent—and (b) from the inverse conditional probability *p*(*T*|*C*)—unless *P*(*C*) and *P*(*T*) are equal. Thus, both the meaning and the value of a conditional probability vary drastically as a function of its reference class^1^.

Our model solves the *mammography problem* by framing a 2 × 2 matrix and focusing on a particular location in a larger framework of probabilistic measures. Before exploring the semantics and labels of additional locations, we should realize that even relatively simple scientific problems are typically not solved by providing a measure and its value (“The PPV is 7.8%.”). Instead, successfully answering a question by deriving a suitable measure is not the end of a scientific enterprize, but the beginning of its dissemination and interpretation. While it is non-controversial that communicating scientific results in a transparent fashion is desirable, explaining what this means and how it can be achieved is far from clear. Interestingly, our model implies a non-circular and non-trivial notion of representational transparency.

### 2.4. Presenting

How can we communicate scientific results in a transparent fashion? For probabilistic measures, the standard solution is to either assume that one's audience is familiar with the measure's definition or to provide it as a mathematical formula. This is perfectly transparent to anyone at ease with the notation and the axioms governing their interpretation, but opaque and intimidating to anyone else. Alternatively, visualizations can be powerful tools for communicating abstract information. While most people agree that most presentations of scientific findings benefit from clear and transparent visualizations (e.g., Tufte, 2001), precisely explaining *why* visualization help remains challenging (see Streeb et al., 2020, for a systematic review). A full-fledged theory of visualizing metrics derived from 2 × 2 matrices is still lacking (though see, e.g., Micallef et al., 2012; Binder et al., 2015, 2020; Khan et al., 2015; Böcherer-Linder and Eichler, 2017, 2019; Eichler et al., 2020, for studies contrasting specific types of visualizations). But as we began this article with Simon's (1981) notion that a problem's solution lies in its transparent representation, we owe an account of what renders representations transparent. Our model suggests a non-circular definition of *representational transparency*:

A representation is *transparent* with respect to a specific task when it explicates the perspective required for solving the task.

When applying this definition to measures derived from a 2 × 2 matrix, we obtain:

A particular measure's representation is *transparent* when it explicates the perspective adopted during the measure's derivation.

Several aspects of these definitions are noteworthy: First, both definitions of transparency are explicitly constrained to a specific task. If this task consists in quantifying some aspect of a 2 × 2 matrix, a transparent representation of the resulting value must explicate the perspective adopted in the measure's derivation. Seeking a more general definition of representational transparency (i.e., beyond the tasks considered in section 2.3 and the measures defined in section 4.2) would need to consider the representation's ecological rationality (see Todd et al., 2012, for details).

Second, the definitions are applicable, but not limited to visualizations. They specifically allow for verbal explications or mathematical notations. Similarly, the definitions are deliberately silent and agnostic about specific types of graphs and the visual feature(s) to which a measure is being mapped. For instance, a measure's numeric value can be expressed by an angle, area, coordinate, or length. Which of those features is most appropriate depends on many factors, including the task to be performed (e.g., does it require a qualitative judgment or a quantitative comparison?), a value's context and magnitude, and the viewer's perception, graph literacy, and motivation.

Third, explicating a measure's perspective typically requires that the measure is being shown, rather than merely being implied by other representational elements. However, merely depicting some measure in a visualization is *not* sufficient for achieving transparency. For instance, mapping the values of probabilities (e.g., accuracy, PPV, or the effects of risks or treatments) to spatial locations or the heights of bars may explicate their numeric magnitude, but provides no information on *how* the values were derived. In fact, visualizations that invite comparisons between non-transparent measures may even obscure and manipulate information, rather than reveal it (see section 5.3 for examples).

**Figure 5:**
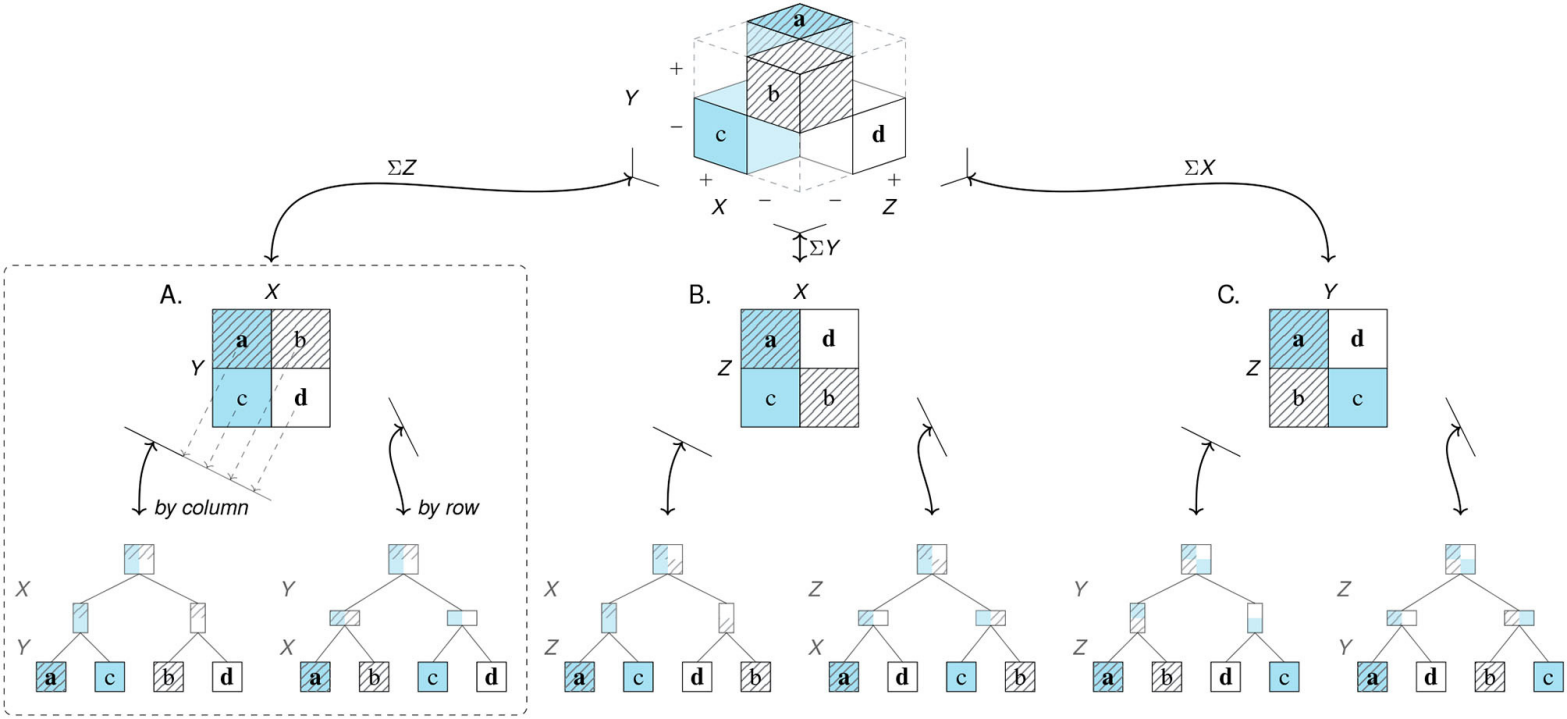
The *partial cube model* shows the geometry of frequency counts resulting from categorizing a population by two binary dimensions *X* and *Y* if a third dimension *Z* expresses the correspondence between *X* and *Y*. Given a population size *N*, the correspondence constraint reduces the full model (containing 2^3^ cells) to four cells (*df* = 3). Arrows are bi-directional and show projections from higher- to lower-dimensional spaces, and vice versa. There exist three distinct 2 × 2 matrices **(A–C)** and six distinct one-dimensional representations (augmented as trees)—all others are mirror images or rotations of these (see Supplement 1 for details). Although all perspectives are informationally equivalent, the dashed region marks the 2D- and 1D-visualizations that are semantically privileged for tasks in which dimension *Z* can remain implicit. (Cell color marks category *X*; pattern marks category *Y*; bold font marks correct classifications *Z*.)

**Figure 6:**
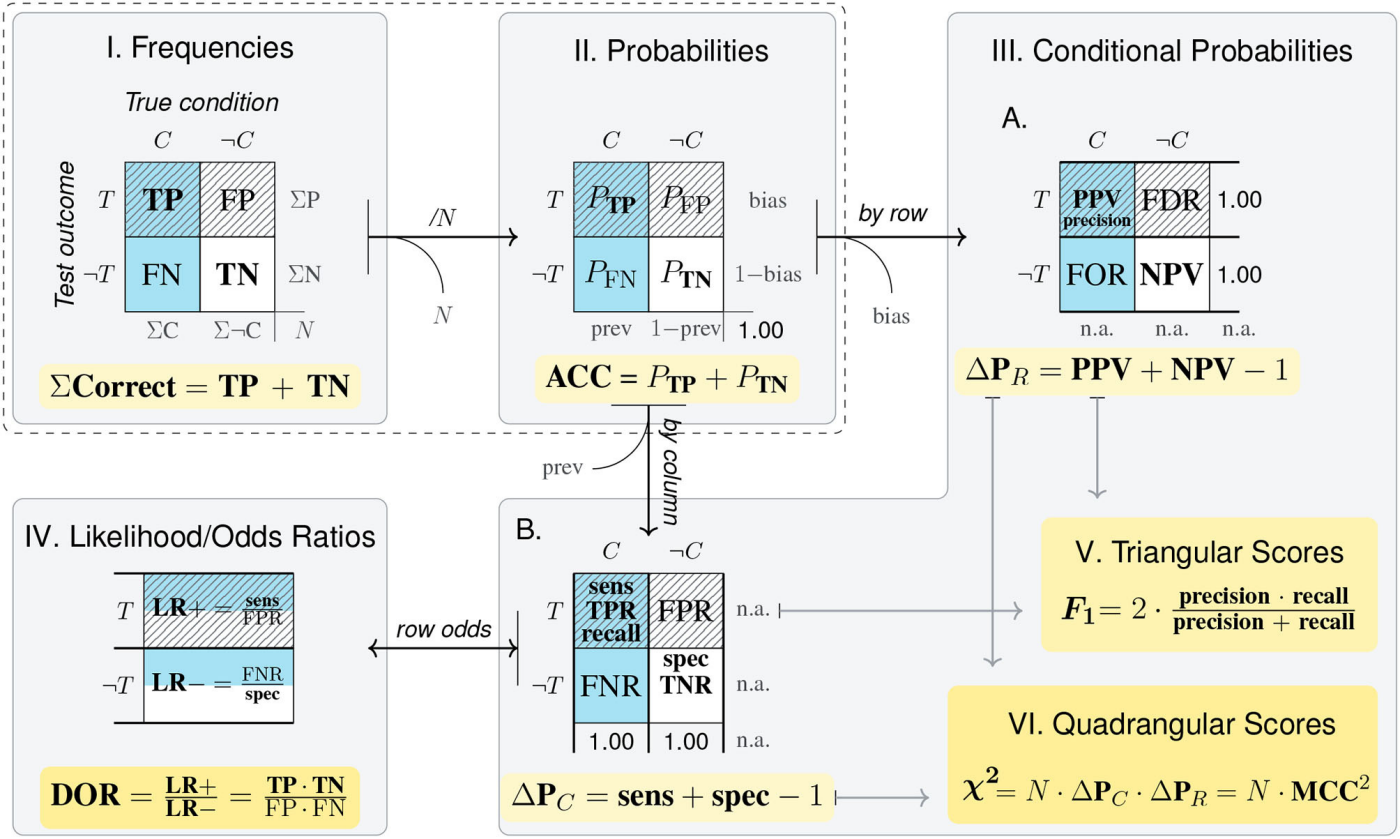
Key metrics for measuring diagnostic classification performance based on a 2 × 2 matrix of frequency counts that denote *true positives* (TP), *false positives* (FP), *false negatives* (FN), and *true negatives* (TN). Panels **I–III** correspond to Figure 4, whereas Panel **IV** computes likelihood and odds ratios from conditional probabilities **(III)** or frequencies **(I)**. The diagram explicates the relations and dependencies between metrics, arithmetic transformations (e.g., normalizing, computing conditional probabilities, or odds), and corresponding changes of perspective. (See Figure 4 for a numeric example and Table 3 for definitions and alternative names.)

How can we explicate the perspectives adopted in the derivation of a particular measure? Although mathematical definitions help explicating how measures are computed, we believe that visualizations are more accessible to a wider audience. Our definition of representational transparency can be read as providing prescriptive guidance, but there is no simple recipe for turning it into a procedure for creating transparent visualizations. Given a vast repertoire of options, we can only provide some examples here. In fact, most of the figures in this article explicate perspectives adopted on a shared representation of a 2 × 2 matrix. For instance, Figure 4 illustrates how probabilities and conditional probabilities are derived from the joint frequencies of a 2 × 2 matrix. In sections 3, 4, we extend this approach to additional visualizations (e.g., hierarchical trees in **Figure 5**) and more complex measures (e.g., of contingency and odds in **Figure 6**). Similarly, the perspectives adopted on a 2 × 2 matrix for deriving the sensitivity or PPV of a diagnostic test can be expressed in the form of an icon. Given the 2 × 2 matrix of the *mammography problem* (shown in Figure 4I), the contrast between the test's *sensitivity* (sens) and its *positive predictive value* (PPV) can be depicted as: sens = 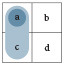 = 80% vs. PPV = 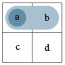 = 7.8%. Although such icons seem suitable for expressing frequencies, probabilities, and conditional probabilities in a compact fashion, they assume a framed 2 × 2 matrix and reach their limits for more complex measures (e.g., the aggregate scores of **Figure 6** or Table 3). Additional options for visually explicating particular perspectives on tasks involving probabilistic measures include *icon arrays, unit squares, tables, tree diagrams*, and *frequency nets* (see Neth et al., 2018, for generating different visualizations from a shared representation, and Binder et al., 2015, 2020, and Böcherer-Linder at al., 2019, 2020 for empirical comparisons).

While this article promotes the matrix lens model as an analytic device, a 2 × 2 matrix may also turn out to be a useful visualization for many problems. For instance, a key structural feature of a 2 × 2 matrix—as an external representation—is that it explicates two orthogonal dimensions. If this also is an important feature of a problem, representing it as a 2 × 2 matrix may facilitate solving it. However, if the task's structure or semantics impose an order on two dimensions, a hierarchical representation (like a unit square or tree) may provide a better fit. Thus, rather than suggesting that the 2 × 2 matrix is the right representation for all problems, we emphasize that evaluating a visualization's degree of fit to a particular task pre-supposes an analysis of the task's structural and semantic aspects. In section 3, we will see that the semantics of many tasks and domains imply a three-dimensional structure. As a consequence, any two-dimensional visualization contains visual artifacts that select and emphasize some aspects while omitting or obscuring others. Although visualizations can be tailored to fit to specific tasks, the downside of any such specialization is a loss of generality. Thus, if problems or domains require transfers between measures or tasks, the costs of tailored visualizations may outweigh their benefits. Overall, the question which visualization fits best for which task—and for which audience—remains an important challenge for future research.

## 3. Semantics

The previous section introduced the matrix lens model as a general approach for solving tasks based on frequency counts, conditional probabilities, and binary contingencies. The model's steps were illustrated by framing specific 2 × 2 matrices of *Titanic* passengers and deriving some measures of the *mammography problem*. However, the model was expressed in abstract terms, involving simple geometric transformations, and a set of basic tasks that could be applied on any population of elements that is filtered into binary dimensions and viewed through the structural construct of a 2 × 2 matrix. Its key mechanism of adopting particular perspectives on this construct derived measures as locations in a matrix-based framework. The meanings of these matrices seemed arbitrary, merely motivated by examples, and did not matter much.

In practice, scientific problems are rarely posed in a semantic vacuum, but rather embedded in specific contexts. As people typically solve problems within particular domains, the concepts and categories used to frame 2 × 2 matrices vary as a function of domain-specific contents. Similarly, the preferred perspectives adopted on 2 × 2 matrices and the terminology of corresponding measures differ substantially between domains.

Semantic questions address issues of meaning, interpretation, and relevance. To clarify semantic sources of confusion in the context of 2 × 2 matrices, we first describe typical task domains and then identify some standard mappings of matrix dimensions and categories in these domains (in section 3.1). Discovering a shared structural feature will then allow us to propose a simplified model that explicates the structure that underlies a range of problems in several domains (in section 3.2).

### 3.1. Mapping Meanings to Dimensions

Due to their structural simplicity, 2 × 2 matrices feature prominently in many tasks and domains. Unfortunately, the commonalities between these uses are obscured by the flexibility in arranging a given 2 × 2 matrix (see section 2.2) and the distinct terminologies of scientific fields (see section 4.2). We use the term *task domain* to denote a discipline or field with a common set of questions and applications. As the questions that can be addressed by a 2 × 2 matrix crucially depend on its dimensions, we characterize task domains by the semantic categories of their typical dimensions.

Table 2 lists the task domains considered in this paper and defines a default mapping of their dimensions. For instance, the *mammography problem* stems from the task domain of *medical diagnostics*. The corresponding 2 × 2 matrix (shown in Figure 4) mapped each patient's *true condition* to *X* and the *test outcome* to *Y*. Table 2 also notes the origins of the matrix dimensions and the dependencies between them (in the rightmost three columns). When using an existing test to diagnose a disease, the *true condition* *X* is given by the environment and the *test outcome* *Y* is given by the test. As discussed in section 2.3, the matrix diagonal represents the correspondence between the other two dimensions. In the context of diagnostics, this correspondence implies the *correctness* of a test result and is listed as a third dimension *Z*.

**Table 2:**
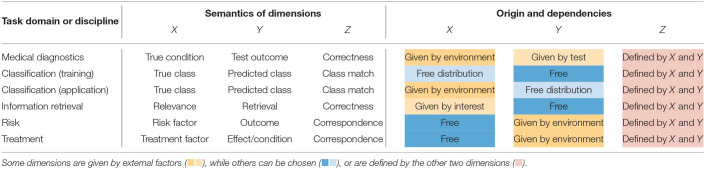
Semantic mappings of concepts to three dimensions of 2 × 2 matrices in different task domains or disciplines.

Beyond medical diagnostics, Table 2 provides default mappings for 2 × 2 matrices of additional task domains that we cannot cover in detail in this paper. In *classification*, the criteria of a *true class* *X* and a *predicted class* *Y* can both be freely chosen by the analyst during training, but the identity of *X* is externally given when applying a classifier. The field of *information retrieval* combines notions from signal detection theory and categorization to search for relevant documents, but uses a distinctive terminology for its metrics (e.g., *precision* vs. *recall*). Its signature task typically implies large numbers of irrelevant documents that are to be ignored (i.e., high values in cell d or joint category ¬*X* ∩ ¬*Y*) as, for instance, expressed in the *null invariance* property by Tan et al. (2004).

The domains of *risk* and *treatment* are similar insofar as both freely set or define the levels of some (independent) Factor *X* and measure or observe the environmental consequences on some (dependent) Factor *Y*. As *treatment effects* are often measured as increases or decreases of medical conditions, such conditions can also be mapped to dimension *Y* of 2 × 2 matrices (resulting in rotations by 90°, relative to the standard 2 × 2 matrix of *medical diagnostics*). Consequently, the referents of the medical terms *prevalence* and *incidence* should always be noted.

Importantly, all domains considered in Table 2 share a structural element: Whereas, the semantic contents mapped to dimensions *X* or *Y* can be chosen freely or are given by external factors, dimension *Z* is *always* determined by *X* and *Y*. Inspecting the semantics of dimension *Z*—noted as “correctness,” “class match,” or “correspondence”—reveals that they all imply some notion of *accuracy*. As a consequence of this regularity, the 2 × 2 matrix {*X, Y*} (i.e., with an implicit dimension *Z*) fits closely to the semantic structure of the task domains considered here. In the absence of a specific task, this particular 2 × 2 matrix is semantically privileged, but some tasks may benefit from an explication of *Z*. Applying the correspondence constraint to a 3D-grid (from section 2.1) yields a modified geometric model that gives rise to more specialized perspectives that explicate particular dimensions and introduce representational constraints.

### 3.2. The Structure of Task Domains

All problems mapped by the task domains of Table 2 correspond to a shared three-dimensional structure. This *partial cube model* (see Figure 5) is created by three orthogonal binary dimensions *X*, *Y*, and *Z*, under the constraint that *Z* represents the correspondence between *X* and *Y*. In contrast to our initial *Titanic* example (in Figure 3), the partial cube model only contains four cells with frequency counts, as four category combinations are rendered impossible by the constraint on *Z* (e.g., the triple *XY*¬*Z* cannot exist). Thus, the partial cube model is fully determined by the frequency counts a, b, c, and d.

As before, viewing the model from the direction of one of its axes collapses the corresponding dimension and frames three distinct 2 × 2 matrices (A–C). Geometrically, adopting one of these perspectives implies a projection from the 3D-model to a 2D-matrix. But due to the fragmentary nature of the partial cube, these projections no longer require any aggregation over the dimension from which it is being viewed. Thus, each of the three possible 2 × 2 matrices fully preserves the frequency information of the 3D-model. Although the three matrices only differ in the arrangement of the four frequency counts, they are not identical. Crucially, each 2 × 2 matrix explicitly represents two of the three original dimensions (as its horizontal and vertical dimensions), whereas the third dimension is implicitly represented (as its diagonal). The 2 × 2 matrix with two orthogonal dimensions {*X, Y*} and an implicit dimension *Z* matches the semantic structure of tasks in which the third dimension is defined as the correspondence of the other two dimensions (as in Table 2). Thus, Matrix A is the most compact 2D-representation that preserves the 3D-structure of the underlying task domain and is semantically privileged over the other matrices, unless a task requires that category correspondence is explicated.

Each 2 × 2 matrix can be organized further by reading out its four cells in either a *by row* or *by column* fashion. Geometrically, this process corresponds to the two possible projections from a 2D-matrix into an ordered list of cells. Collapsing a matrix into a list is also known as stacking dimensions (Mihalisin et al., 1991), and can be augmented as a hierarchical tree that illustrates how each matrix is parsed into the ordered sequence formed by its leaves. Depending on the angle from which a matrix is being viewed, the projection results in two distinct trees and lists per matrix: The left tree below each matrix uses the horizontal dimension as the tree's first branching criterion (i.e., dissecting the matrix in a *by column* fashion) before using the vertical dimension as the tree's second branching criterion (dissecting the cells of each column *by row*). The right tree below each matrix assumes a different projection angle, thus reversing the branching criteria of the left tree and reordering the list's four frequency counts into a different order as the tree's leaves. The six trees and lists at the bottom comprise all possible ways of projecting the original frequency counts into one-dimensional lists (see Supplement 1 for details).

To clarify the status of the geometric model shown in Figure 5, note that the top cube explicates the actual structure underlying any task with semantic mappings that define one dimension as the correspondence between two others (i.e., dimension *Z* in Table 2). More precisely, the image of the partial cube provides a visualization of this structure, but its geometry models the essential aspects of tasks with three orthogonal dimensions and the correspondence constraint. By contrast, all lower-dimensional visualizations (e.g., the 2 × 2 matrices and trees in Figure 5) selectively depict some particular aspect of this structure. Depending on the current task, such visualizations can both increase and decrease the transparency of particular measures (see section 2.4). As the discovery of a shared representational structure has the potential to integrate the terminologies and metrics used in many different domains, it is important to understand in which sense the representations on the three levels of Figure 5 are identical to and differ from each other. On the one hand, all ten images contained in Figure 5 are *informationally equivalent* (Larkin and Simon, 1987). In contrast to the projections in Figure 3, every 2 × 2 matrix, hierarchical tree, or list of counts contains the frequency information of the original cube, and thus can be reconstructed from any other image. (Supplement 1 shows that the three-, two-, and one-dimensional models enable an identical number of 24 distinct projections.) On the other hand, this does not imply that all these images are equal. Instead, they differ substantially in the ways in which they explicate and organize information. Strictly speaking, only the partial 3D-cube faithfully represents the three-dimensional nature of the underlying problem. By adopting particular perspectives, all two- or one-dimensional projections distort this structure by imposing new constraints and introducing representational artifacts that can have both desirable or undesirable consequences, depending on the task to be solved. For instance, framing a 2 × 2 matrix by selecting and arranging two dimensions not only renders the third dimension implicit, but also alters the proximity relations between cells (as some become neighbors, while others are separated). Similarly, whereas the original cube contains no hierarchy, each tree depicts one dimension as the primary and unconditional branching criterion (dissecting the population into two subsets) and one other dimension as a second branching criterion (appearing to be dependent and conditional upon the first). Importantly, the structure of a matrix or tree is blind to all semantic constraints of specific tasks or domains. Thus, a chosen representation neither needs to correspond to a user's current task (e.g., a 2 × 2 matrix of *X* by *Y* can be shown to ask questions about *Z*), nor match the causal or statistical properties of a domain (e.g., the second branching criterion of a tree can be independent of its first). As mismatches between the properties of tasks and representational features make problems more difficult, whereas matches can render solutions transparent, it matters which particular representation is chosen. (We elaborate on this point in section 5.)

## 4. Integration

We originally motivated the matrix lens model by the *mammography problem* and showed how it can be solved by framing and focusing on parts of a 2 × 2 matrix (see section 2). We then added semantic mappings to an abstract model and argued that tasks in various domains share the same underlying structure (section 3). However, both the matrix lens model (shown in Figure 2) and the reduced structural geometry of the partial cube model (Figure 5) seemed ill-motivated if they only allowed to compute the PPV of this particular problem. To justify our investment, we now extend the scope of our model in two ways: First, we show how additional measures of clinical diagnostics can be derived by adopting slightly different perspectives on the same matrix. Locating these measures in our structural account also allows illuminating two key dichotomies in the context of diagnostic testing. In section 4.2, we further generalize our model to additional domains and show how a large variety of measures and terminologies can be understood in a matrix-based framework.

### 4.1. Integrating Metrics of Classification and Diagnostics

Our model solved the *mammography problem* by adopting a particular perspective on a 2 × 2 matrix to derive a test's PPV (Figure 4). As the geometry of the matrix and the abstract tasks performed with this construct are independent of a particular content, we can generalize our analysis to other situations involving classification tasks and diagnostic tests. Figure 6 provides a glimpse of the additional measures that are available by framing and focusing on particular aspects of a 2 × 2 matrix. Figure 6 uses the same layout as Figure 4, but replaces the four frequencies a, b, c, and d, by the nomenclature of *true positives* (TP), *false positives* (FP), *false negatives* (FN), and *true negatives* (TN), which is widely used in the domain of classification and clinical diagnostics. As before, Figures 6I–III show frequencies, probabilities, and conditional probabilities, but Figure 6IV adds *likelihood ratios* (*LR*+ and *LR*−) as row-wise ratios of the conditional probabilities in Figure 6IIIB. The highlighted formulas below each matrix compute metrics that summarize its quality in different ways: By computing the diagonal total of correct cases, *accuracy* (ACC), or two measures of *contingency* as the difference between conditional probabilities in a particular direction (ΔP_*R*_ vs. ΔP_*C*_). A noteworthy aspect of Figure 6 is that some conditional probabilities (in Figures 6IIIA,B) are not only labeled as “rates” (e.g., TPR, FPR), but carry dedicated names (e.g., sens, spec, PPV, NPV) or even multiple names (e.g., sens ≅ *recall*, PPV ≅ *precision*). As we will see in Table 3, this reflects their role and relevance in various domains. But irrespective of semantics, Figure 6 shows dependencies in a diagrammatic fashion. For instance, by conditionalizing the 2 × 2 matrix *by row*, all values of **Figure IIIA** (e.g., PPV, NPV) depend on a condition's *prevalence* (prev), but not on a test's *bias*. Conversely, by conditionalizing the 2 × 2 matrix *by column*, all values of Figure 6IIIB (e.g., sens, spec) depend on *bias*, but not on *prevalence* (prev).

**Table 3:**
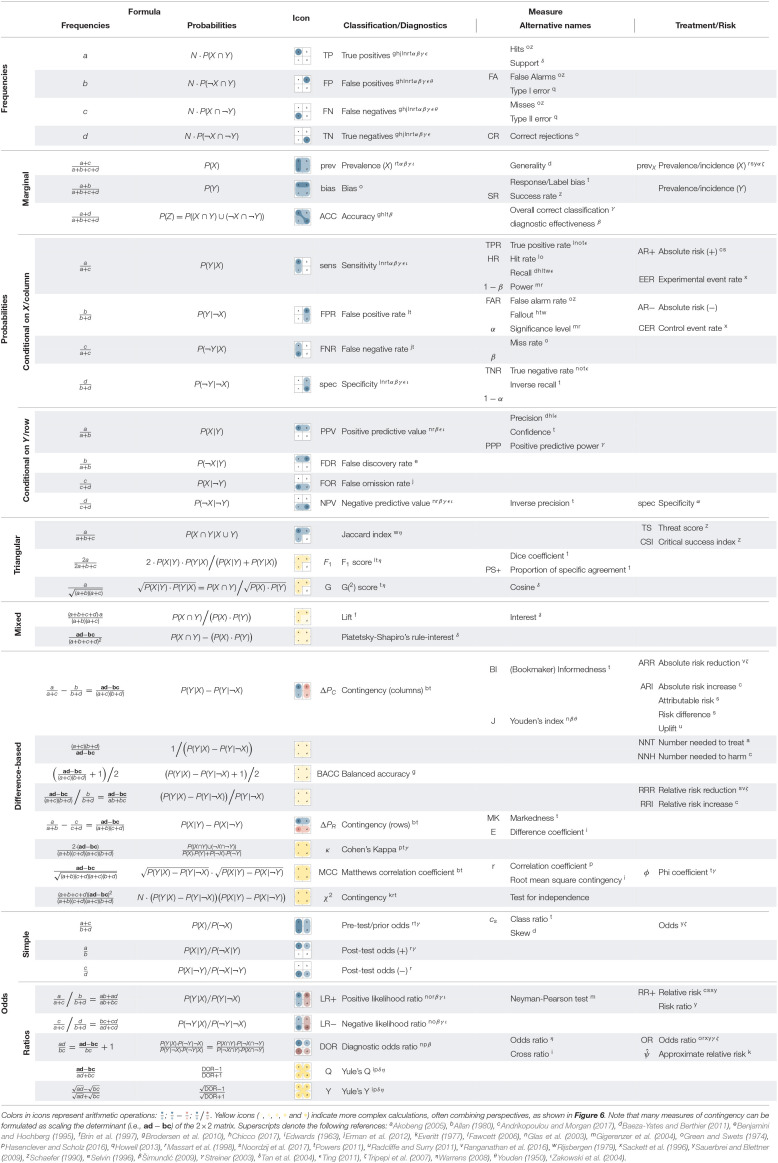
Definition of metrics and corresponding formulas based on the 2 × 2 matrix, and alternative names in different domains or disciplines.

In addition to the familiar frequencies, probabilities, and conditional probabilities, Figure 6 defines three more comprehensive measures that further combine and transform conditional probabilities. The *diagnostic odds ratio* (DOR, defined in Figure 6IV) is a global indicator of discriminative performance that allows comparisons between diagnostic tests (see Glas et al., 2003; Šimundić, 2009, for details). Whereas, its formula implies that it integrates all four elementary frequencies of the 2 × 2 matrix, the geometry of Figure 6 shows that its value depends on a test's *sensitivity* (sens) and *specificity* (spec, both in Figure 6IIIB), but decidedly *not* on a condition's *prevalence* (prev, Figure 6II), as this information was dropped when adopting a *by column* perspective on the original matrix before calculating the likelihood ratios^2^.

Additionally, the lower right panels of Figure 6 define two bi-directional scores that reintegrate the two perspectives adopted by computing conditional probabilities (in Figures 6IIIA,B). The *F*_1_*-score* is the harmonic mean of *precision* (i.e., PPV) and *recall* (i.e., sens) and is called *triangular* (in Figure 6V) as it focuses on the top-left cell and combines two measures that conditionalize the number of *true positives* (TP) both *by row* and *by column*. The χ^2^*-score* (Figure 6VI) is even more encompassing by multiplying both directional measures of contingency (i.e., ΔP_*R*_ and ΔP_*C*_) and additionally including the population size *N*, which otherwise is lost when transforming into probabilities. Finally, the same panel also mentions the popular *Matthews correlation coefficient* (MCC) as another *quadrangular* measure closely related to the χ^2^-score.

Introducing these measures within a structural model of 2 × 2 matrices—rather than using mathematical notation—has two advantages: First, visually illustrating the perspectives adopted by the measures and separating them from the numerical transformations required for their derivation highlights their dependencies and limitations. For instance, realizing that diagnostic situations usually imply a trade-off between two different errors (i.e., incorrect classifications FP vs. FN), Figure 6 visually explains the inverse relationship between *sensitivity* and PPV (i.e., recall and precision). Second, explicating the perspectives adopted by otherwise abstract measures and locating them within a structural framework increases their transparency and facilitates their understanding.

The distinction between adopting two perspectives on a 2 × 2 matrix also helps explaining two key dichotomies in the domain of clinical diagnostics. First, developing a new test adopts a different perspective than applying an existing test (Linn, 2004). *Developing* a test assumes that each element's true condition (and hence the condition's prevalence in the population) is known. Based on this assumption, developers adopt a *by column* perspective and aim to design a test that meets certain criteria, typically formulated in terms of sensitivity and specificity. By contrast, *applying* an existing test assumes that the test's properties are known (as in the *mammography problem*). Based on this information, we can ask questions about the predictive power of a test result. But in order to adopt the corresponding *by row* perspective (e.g., for computing the test's PPV or NPV), we need an actual prevalence value (which may diverge from the prevalence value assumed during test development).

An ideal test would exhibit perfect sensitivity and perfect specificity. But given that we typically need to compromise between both measures, shifting perspectives on the 2 × 2 matrix also illuminates the difference between testing for screening vs. for diagnostic purposes (Morrison, 1998; Streiner, 2003; Trevethan, 2017). In *screening* an entire population, our primary goal is to reliably detect all diseased individuals (i.e., rule out only healthy individuals, Zakowski et al., 2004). Assuming that the prevalence of the condition is low and we have options for further testing, this implies maximizing *sensitivity* (sens) by minimizing misses (FN), at the expense of accepting some false positives (FP). Adopting an alternative *by row* perspective on the 2 × 2 matrix resulting from such a screening scenario, we realize that minimizing misses (FN) at the expense of false positives (FP) will increase the test's NPV, at the expense of lowering its PPV. By contrast, *diagnostic* testing typically starts with a suspicion (e.g., the presence of symptoms or a positive test result) and assumes a higher prevalence of disease. Here, our primary goal is to avoid unnecessary treatments by reliably identifying all healthy individuals (i.e., rule in only diseased individuals, Zakowski et al., 2004). This implies maximizing *specificity* (spec) by minimizing false positives (FP) at the expense of accepting some misses (FN). Viewing the resulting 2 × 2 matrix from a *by row* perspective shows that this will increase a test's PPV at the expense of lowering its NPV. In practice, additional factors—like differences in costs, prevalences, and the availability of other tests or treatments—will also matter. Importantly, our model helps rendering these theoretical relationships more transparent.

### 4.2. Integrating Metrics and Terminologies Across Domains

Beyond the realms of classification and diagnostics, the 2 × 2 matrix construct features prominently in many additional contexts and domains. While many authors have provided overviews that define and summarize the measures used within a domain, few have explained and linked measures across domains. When realizing that an impressive wealth of important measures is based on the relatively simple construct of a 2 × 2 matrix, the lack of an integrative account is striking and calls for an explanation. We see three obstacles and corresponding sources of confusion:

1. First, any attempt to bridge domains faces *terminological* difficulties. For instance, authors from clinical diagnostics (e.g., Selvin, 1996; Massart et al., 1998; Šimundić, 2009) use different names for the same concepts than those rooted in signal detection theory (e.g., Green and Swets, 1974; Stanislaw and Todorov, 1999) or those from machine learning and information retrieval (e.g., Rijsbergen, 1979; Fawcett, 2006; Baeza-Yates and Berthier, 2011; Powers, 2011; Ting, 2011).
2. Domains differ in their *conceptual* needs and thus develop and use different metrics. Whereas, experts in medical diagnostics primarily focus on the conditional probabilities and odds ratios discussed in section 4.1 (see Figure 6), the merits of triangular scores—like the *F*- and *G*-scores, *lift*, or the *Jaccard* index—mainly matter in the context of classifier development and information retrieval tasks (e.g., Rijsbergen, 1979; Powers, 2011).
3. A subtle but pervasive barrier to an integrative account is of a *functional* nature: Whereas, most domains mentioned so far primarily address some variant of a classification task (e.g., “Which of two classes does an individual belong to?” or “What would be a good criterion to distinguish between these two categories?”), the domains of *risk* and *treatment* primarily evaluate the consequences of some categorical distinction (e.g., “Which outcomes are observed if the risk factor is present?” or “What are the effects of being treated?”). Although such questions can readily be addressed in a 2 × 2 matrix framework, the corresponding research traditions differ substantially in their constraints and study designs. Importantly, the usefulness of any particular measure cannot be determined solely from its formula or label, but depends on boundary conditions. An example is the measure of *relative risk* (RR), which corresponds to the *positive likelihood ratio* (LR+) defined in Figure 6: RR can be a useful measure for comparing the outcomes for individuals exposed to some risk factor to those of unexposed individuals (Sauerbrei and Blettner, 2009), a deceptive and misleading measure that inflates the absolute magnitude of effects (Gigerenzer et al., 2007; Noordzij et al., 2017), or an un-informative and nonsensical measure if the risk factor's prevalence was fixed by the study design (Sauerbrei and Blettner, 2009). Thus, choosing and using measures in a sensible manner requires more than just knowing their names and definitions—it requires understanding their roles in answering particular questions and their match to the study design that generated the 2 × 2 matrix.

Despite these obstacles, Table 3 provides an overview of metrics across domains. Previous accounts mostly focused on covering one domain (see, e.g., Hasenclever and Scholz, 2016, for a mathematical/statistical approach, or Todeschini et al., 2012, for an extensive comparison from a bio-chemical point of view) or on connecting two domains (e.g., Powers, 2011). By contrast, our model integrates a wide variety of measures from different domains in a uniform approach and provides—to the best of our knowledge—the most encompassing account so far. Beyond satisfying an encyclopedic ambition to collect key measures from different domains in one place, Table 3 organizes them in a systematic fashion and links various domains and terminologies.

Overall, successful focusing on a single measure reduces the complexity of the world to a one-dimensional answer (see Figure 2). As we have seen, any measure provided as such an answer is a highly specialized tool that—given precise boundary conditions—serves particular purposes. By abstracting from the original data and combining many aspects, the more complex measures gain generality, but simultaneously obscure and encapsulate the perspectives adopted during their derivation.

Besides defining each measure in terms of frequencies and probabilities, Table 3 also provides visual icons that show the perspective adopted on a 2 × 2 matrix when deriving the measure and thus implicitly contained in it. We trust that readers will find these visual and diagrammatic illustrations more illuminating than a purely mathematical treatment. Ideally, locating measures and their inter-relations in a shared 2 × 2 matrix framework will facilitate their comprehension and, hopefully, help to choose and use them more responsibly. To illustrate how the 2 × 2 matrix construct can clarify theoretical debates, the next section applies our approach to some problems that are known to puzzle and perplex people when expressed in conventional form.

## 5. Applications

Our model views the world through the lens of a 2 × 2 matrix. Being a theoretical framework, its primary purpose is to enable insights by explicating the process that reduces selected aspects of a complex and continuous world to a numeric measure. Whereas, such measures are typically defined in terms of mathematical formulas, our structural account reveals them as particular perspectives on a 2 × 2 matrix. Showing how the measures of different domains are based on a common construct and a shared set of basic tasks allows an integrative view of their assumptions and terminologies.

Beyond a better understanding of theoretical concepts and their relations, a practical benefit of our model lies in its potential for clarifying familiar problems. In the following, we provide three case studies that demonstrate how our model can be applied to ongoing debates regarding the difficulty and facilitation of Bayesian reasoning tasks (sections 5.1, 5.2), and to address the question whether the women and children of the *Titanic* were successfully rescued first (section 5.3). True to its analytic nature, our model will not solve these debates, but increase transparency by providing alternative perspectives.

### 5.1. Perspectives on Natural Frequencies and Nested Sets

How can we render the *mammography problem* more transparent? We argue that our model makes three inter-related contributions that help to clarify the theoretical debate surrounding this problem. First, we provide a representational explanation of the problem's difficulty. As we have shown (in sections 1.2, 2.3), the *mammography problem* revolves around three conditional probabilities: Whereas, the test's sensitivity *p*(*T*|*C*) and false positive rate *p*(*T*|¬*C*) are given, the problem asks for the test's PPV *p*(*C*|*T*). When arranging the problem's joint frequencies or probabilities in a 2 × 2 matrix (as in Figures 4, 6) we see that the two conditional probabilities provided adopt a *by column* perspective on the matrix (Figures 4IIIB, 6IIIB), whereas the problem's solution requires adopting a *by row* perspective on the same matrix (Figures 4IIIA, 6IIIA). Geometrically, the problem requires the *reversal* of an adopted perspective before adopting an alternative perspective. Mathematically, providing the prevalence *p*(*C*) renders the reversal possible (i.e., we can re-construct Panel II from Panel IIIB). In practice, however, this requires first computing two joint probabilities [i.e., *p*(*C* ∩ *T*) = *p*(*C*)*p*(*T*|*C*) and *p*(¬*C* ∩ *T*) = *p*(¬*C*)*p*(*T*|¬*C*)] before Bayes' theorem can be used to compute the desired solution *p*(*C*|*T*). Thus, within our 2 × 2 matrix framework, the crux of the Bayesian inversion task are its *representational* demands, which are reflected in its computational complexity. Even when fully understanding the information provided and the question asked, solving the standard *mammography problem* requires two representational shifts: Reversing an implicit perspective and pivoting to an alternative perspective.

**Figure 7:**
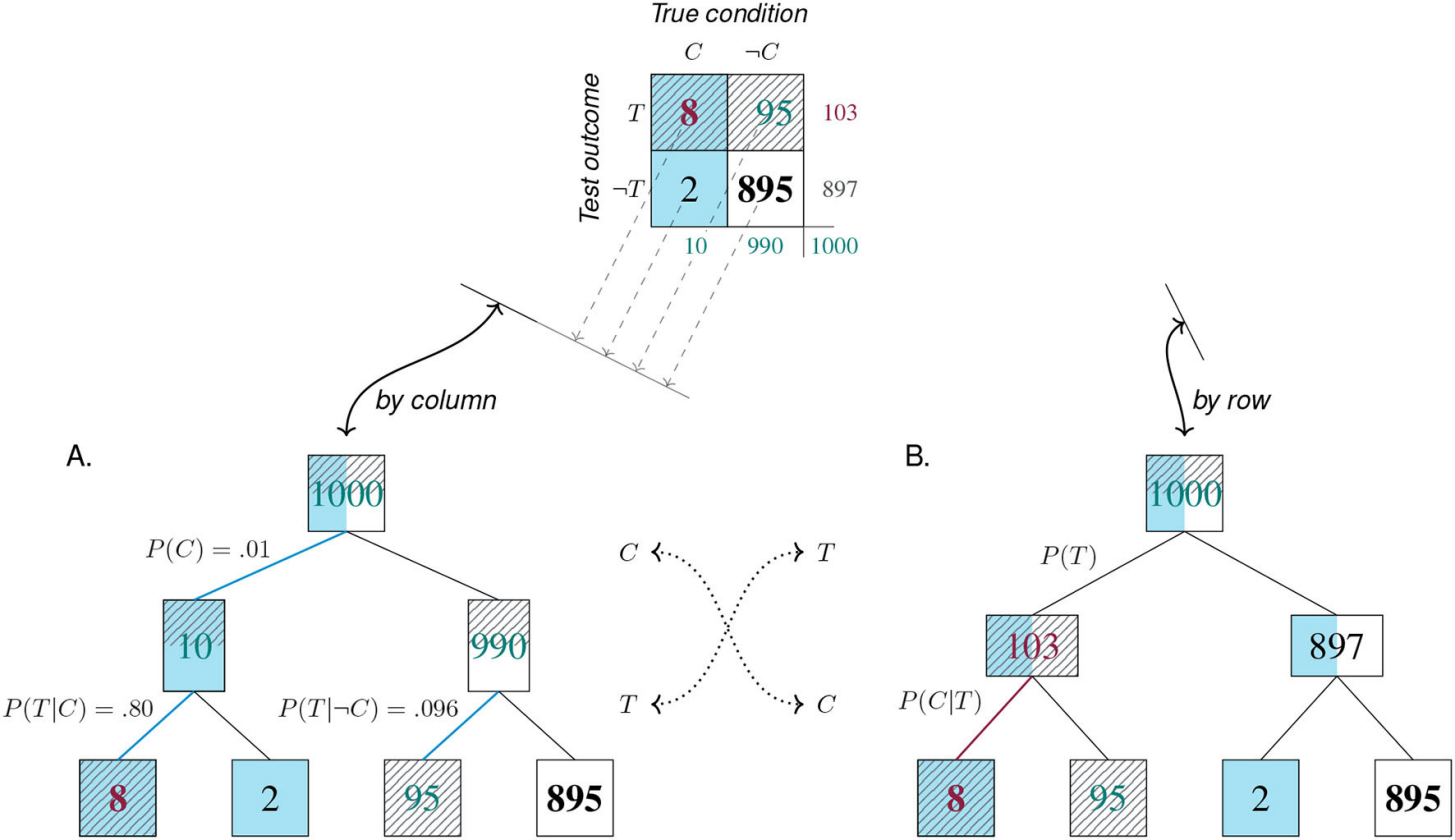
A 2 × 2 matrix of the *mammography problem* and two trees resulting from adopting a *by column* vs. a *by row* perspective on it. Computing the PPV from natural frequencies for a population of *N* = 1, 000 women requires realizing that 8 out of 103 women with a positive test result also have cancer. Irrespective of its format, the information provided by the Bayesian problem (probabilities in blue, frequencies in green or red) only allows the direct construction of Tree **A**. However, only Tree **B** explicitly represents both frequencies required for solving the task (in red). Thus, while Tree **A** provides a transparent representation of the problem, Tree **B** renders its *solution* transparent.

As a second contribution, our model partially explains why expressing the problem in the standard frequency format makes its solution much easier. We propose two representational reasons for the facilitative effect of natural frequencies on Bayesian inference. First, let us assume that the four basic frequencies (a–d) are framed as a 2 × 2 matrix (as in Figures 4I, 6I). Given this matrix, the desired PPV *p*(*C*|*T*) can be derived in a straight-forward manner—by focusing on the top row (i.e., women with a positive test result *T*) and computing the ratio $\frac{\text{a}}{\text{a}+\text{b}}$. Arithmetically, this operation is identical to the computationally simple solution based on a natural sampling process (e.g., Gigerenzer and Hoffrage, 1995; Hoffrage et al., 2000, 2002). Comparing the representational complexity of this process to the one outlined for the probability format reveals a stark contrast: Instead of reversing an implicit perspective before switching to another, we only need to adopt a single *right* perspective on the 2 × 2 matrix. But what if natural frequencies are *not* already framed neatly in 2 × 2 matrix form? Interestingly, assuming the absence of a 2 × 2 structure may render the adoption of the right perspective even easier. Our second representational reason for the higher likelihood of correct solutions when expressing the problem in the standard frequency format considers the identities and semantics of the joint frequencies provided. Note that the problem statement explicitly provides only two of four joint frequencies: a = 8 and b = 95. The semantic category shared by these frequencies is *T* (i.e., women with a positive test outcome). Noticing this common element is the mental equivalent of adopting a *by row* perspective on the 2 × 2 matrix, or constructing an hierarchical tree that uses the *Test outcome* dimension as its first branching criterion. (As we will see in Figure 7B, adopting this perspective essentially solves the problem.) Thus, framing the joint frequencies as a 2 × 2 matrix facilitates the solution by requiring fewer perspective changes than starting from two conditional probabilities and a prior. And providing only the two joint frequencies that need to be combined for deriving the correct solution may even act like a mental nudge into the right direction.

Given abundant evidence for the facilitative effects of natural frequencies on Bayesian reasoning, a puzzling finding from decades of research is that about 75% of the participants facing such problems still *fail* to provide the correct solution (McDowell and Jacobs, 2017). Thus, a very good question (raised by Weber et al., 2018) is: Why is Bayesian reasoning in frequency formats still so difficult? Our third contribution builds on the previous two and provides an analytic answer to this question. As we have seen, the *mammography problem* in its standard probability format provides sufficient information for applying Bayes' theorem or for translating the problem into an alternative representation using natural frequencies. By specifying the cancer prevalence *p*(*C*), the test's sensitivity *p*(*T*|*C*), and its false positive rate *p*(*T*|¬*C*), the three measures typically provided adopt a *by column* perspective on a 2 × 2 matrix framed by *True condition* as its Dimension *X* (see Figures 4, 6). As a consequence, reconstructing the frequency matrix from the probabilities provided implies building a hierarchical tree that first dissects the population by *True condition* before branching by *Test outcome* (see Tree A of Figure 7, which shows provided probabilities as blue edges). Importantly, expressing the problem in the standard frequency format provides five key nodes of the *same* tree (in green and in red). Thus, although the underlying problem structure actually enables three 2 × 2 matrices and six hierarchical trees (see Figure 5), the only tree that can directly be constructed from the provided information splits the population by *True condition* (i.e., adopts a *by column* perspective on the matrix). By contrast, the PPV measure solving the problem adopts a *by row* perspective on the same matrix. Hence, instructing a representation of Tree A for computing the PPV still requires a change in perspective: Rather than combining tree leaves by *True condition*, they must be combined by *Test outcome* (to see that the number of women with positive tests is 8+95 = 103). Making this change effectively constructs an alternative tree that corresponds to adopting a *by row* perspective on the 2 × 2 matrix (see Tree B of Figure 7, which explicitly represents both frequencies required for computing the PPV in red). Importantly, *both* trees are perfectly transparent, but with respect to different tasks. Both standard formats instruct Tree A which transparently represents the information provided by the *problem*. The task remains difficult because its solution is not obvious in this representation—only Tree B adopts the perspective required for deriving the PPV and thus provides a transparent representation of the task's *solution*. Thus, our geometric analysis shows that Bayesian reasoning is and remains vexing as long as it requires a crucial representational shift between problem statement and solution. Even when expressing the Bayesian problem in terms of natural frequencies, the perspective implicitly adopted by the provided information has problem solvers, metaphorically, and literally, barking up the wrong tree. Taking (Simon, 1981) seriously, we suggest: By making the problem's solution transparent, the right tree solves the problem.

Accepting this insight raises an intriguing conundrum: If the crux of Bayesian problem solving consists in the representational shift, what remains when we provide people with a transparent representation of the solution? Removing the need for a perspective change essentially *dissolves* the Bayesian aspect of the original problem^3^. Thus, it should not surprise us that providing participants with the crucial elements of Tree B (as in the short menu formats by Gigerenzer and Hoffrage, 1995) or both trees (as in the double tree by Wassner, 2004) improves the likelihood of correct solutions. What *should* surprise us, however, is that their rate fails to reach 100%. Based on our representational analysis, instructing the problem in a short menu format (or one of its visual analogs) essentially tests participants' ability to recognize the solution when its key elements are provided to them. As the term “facilitation effect” seems misleading in the absence of a Bayesian problem, it may be more appropriate to view this experimental condition as providing an upper performance benchmark (in the sense of Neth et al., 2016), which assess people's ability or willingness for deriving and reporting a conditional probability when the representational demands of the Bayesian problem have been removed. The empirical finding that the solution rates in conditions with short menu formats only rise by about 12% (McDowell and Jacobs, 2017) suggests that participants suffer from additional difficulties that prevail beyond the representational demands of Bayesian reasoning (e.g., lack of comprehension, motivation, or numerical skills. See Brase, 2009a; Ferguson and Starmer, 2013; Weber et al., 2018, for suggestions).

**Figure 8:**
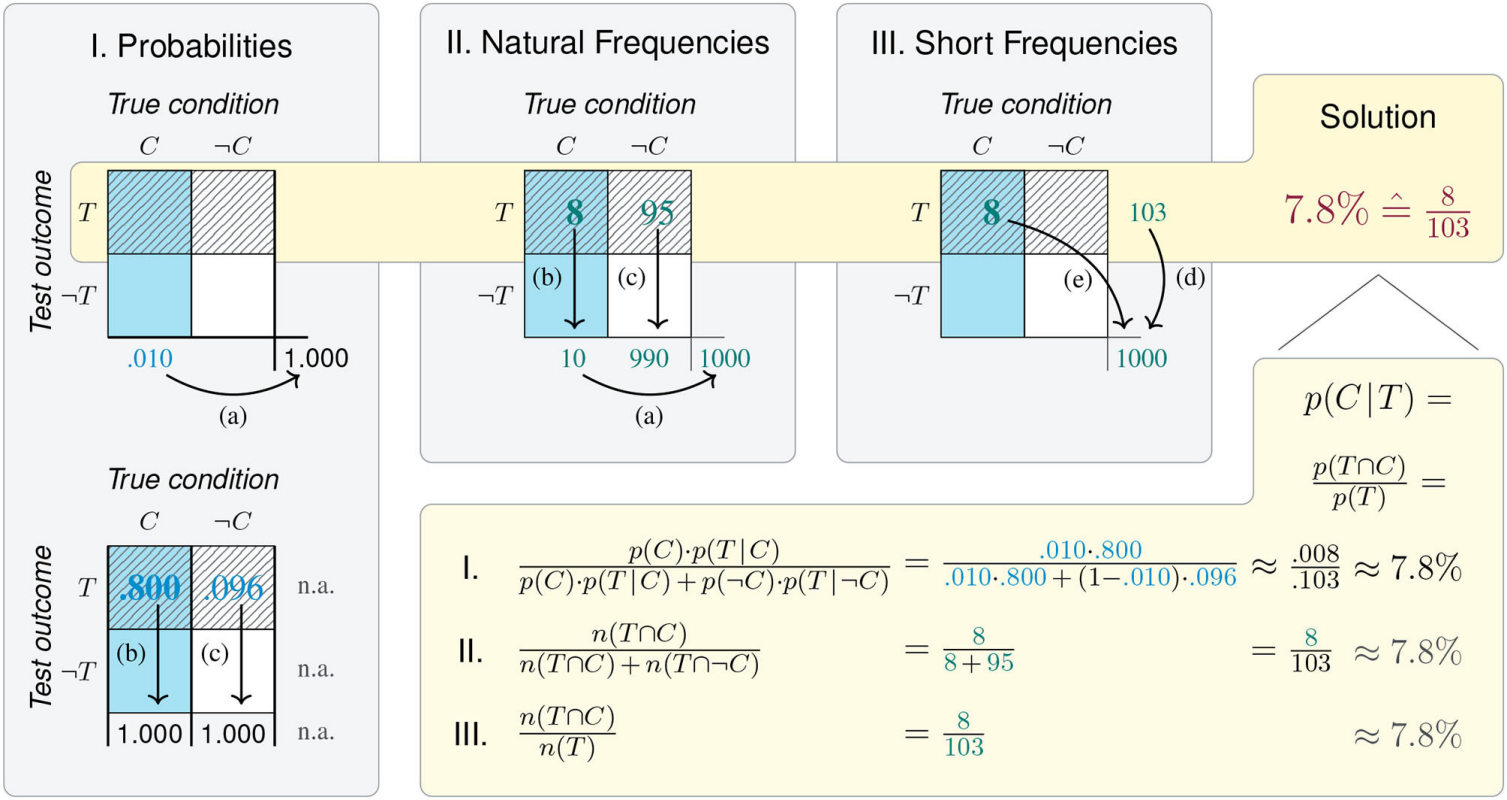
A matrix-based account explicating the difficulty of Bayesian reasoning and two types of facilitation effects (on the example of the *mammography problem*, as studied by Gigerenzer and Hoffrage, 1995, p. 688). (Color coding and lowercase letters next to arrows in Panels **I–III** refer to the problem descriptions in the three formats shown in Table 1.)

Figure 8 summarizes our arguments on the representational demands of Bayesian reasoning and the facilitation effects of natural frequencies and short menu formats. Beyond the computational differences (shown in the lower right panel), the information provided by the problem and the perspectives required and suggested for solving it differ substantially between the three problem versions. The probability format (Figure 8I) mixes a marginal probability and two conditional probabilities that both adopt a *by column* perspective. The two joint probabilities of the 2 × 2 matrix containing probabilities (marked as missing parts of the Solution in Figure 8) are necessary for solving the problem, but first need to be computed from the probabilities provided. The natural frequencies format (Figure 8II) presents information in the same (*by column*) perspective as the probability format (as indicated by the vertical arrows), but provides frequencies instead of probabilities. Reducing this difference to a mere change in representational format ignores the representational differences between both panels. Figure 8II renders it obvious why the problem's solution is facilitated: The two joint frequencies that are explicitly mentioned in the problem are also required for computing its solution and suggest the right *by row* perspective. Finally, the short frequencies format (Figure 8III) abandons the *by column* perspective of the other panels. By providing a joint and a marginal frequency, the alternative *by row* perspective is suggested and implies the solution. Especially if the answer asks for frequencies (i.e., 8 out of 103), the short frequency format essentially becomes a search task that does not require any calculation.

To clarify, our representational account does not compromise the key argument of Gigerenzer and Hoffrage (1995), who demonstrate the facilitative effects of frequency formats on Bayesian reasoning. But whereas previous authors saw the benefits of short menu formats primarily in reducing computational complexity (e.g., Ferguson and Starmer, 2013; Fiedler et al., 2000; Mellers and McGraw, 1999), we argue that removing the need for a perspective change fundamentally alters the problem. Whereas, natural frequencies only facilitate performance by implying a more goal-directed representation of the Bayesian problem, the short menu format suggests this alternative perspective, thereby explicating the problem's solution in a transparent fashion. Despite these contributions, any attempt to explain all existing data solely on the structure of a 2 × 2 matrix would inevitably fall short, as its geometry remains silent about the difference between joint frequencies and joint probabilities (i.e., Figures 4I,II, 6I,II). Studies demonstrating the impact of representation formats (e.g., Sedlmeier and Gigerenzer, 2001; Brase, 2008) and the relevance of analytical abilities (e.g., Sirota et al., 2014) show that representation format, problem content and context, and individual differences jointly matter for performance in Bayesian reasoning.

Our analysis has both theoretical and practical implications for investigations of Bayesian reasoning. Theoretically, our account is compatible with the basic tenets of *nested-sets theory*, which claims that Bayesian inference is facilitated by rendering certain subset relations and their reference classes more transparent (e.g., Mellers and McGraw, 1999; Sloman et al., 2003; Yamagishi, 2003; Barbey and Sloman, 2007). But advocates of nested-sets theory have been criticized that “the mechanism by which the subset structure is revealed has not been specified. Nor is it clear how the joint event formats help participants to visualize the nested structure.” (McDowell and Jacobs, 2017, p. 1293). By contrast, our model provides concrete suggestions how specific sets are made accessible (by *filtering* and *framing*) and how subset structures are revealed (by *focusing* on different parts of a shared representational structure). In fact, our notion of adopting particular perspectives provides a mechanism that explains why some formats or menus facilitate the problem's solution more than others: Given a 2 × 2 matrix, both natural frequencies and short menu formats enhance the salience of the perspective that renders the problem's solution transparent. Various authors have expressed similar ideas—see, for instance, the notion of *backward reasoning* by Johnson and Tubau (2015), the *problem-representation transfer hypothesis* by Sirota et al. (2015), or ideas on the importance of *task-compatible reference classes* by Ayal and Beyth-Marom (2014) and Talboy and Schneider (2018)—but anchoring their hypotheses in a structural account makes these notions more specific and concrete. Finally, the apparent discord between *natural frequencies* and a *nested-sets* account dissolves within our model: Natural frequencies are an implicit result of *filtering* and *framing* (see sections 2.1, 2.2). Nested-sets theory describes how natural frequencies are selected and explicated, which our model depicts as particular ways of *focusing* (section 2.3).

As a practical implication, our representational account appoints a key role to the systematic study of visualizations for improving Bayesian reasoning. Researchers in both visualization (e.g., Cleveland and McGill, 1985; Ziemkiewicz and Kosara, 2010) and psychology (e.g., Talboy and Schneider, 2017; Böcherer-Linder and Eichler, 2019) agree that proportional visual mappings are essential for providing useful visual aids. However, our analysis suggests that experimental designs should move beyond comparing performance with and without visual aids (e.g., Brase, 2009b; Garcia-Retamero and Hoffrage, 2013) or contrasting seemingly haphazard selections of graphical representations (e.g., Micallef et al., 2012; Khan et al., 2015). As a comprehensive study of visualizations for Bayesian reasoning is still lacking, existing classifications of visual representations are typically described as collections of examples (e.g., Binder et al., 2015, Figure 1, p. 3; McDowell and Jacobs, 2017, Figure 2, p. 1283; and Böcherer-Linder and Eichler, 2019, Figure 3, p. 3). Although some noteworthy structural accounts of visualizations exist (e.g., Khan et al., 2015; Böcherer-Linder and Eichler, 2017, 2019), they were mostly framed in terms of nested-sets. Lacking the mechanisms of adopting particular perspectives on a shared representation, they could not benefit from the three-dimensional structure underlying all Bayesian reasoning problems (see Figure 5) or justify why some representations are privileged, while others are misleading. As we have shown (in sections 2, 3), contrasting different visualization types risks comparing apples with oranges (e.g., a 2 × 2 matrix with two optional perspectives, with the particular perspective of a tree or unit square). To be aware of such categorical distinctions, we must always specify: Which particular version of each visualization is being shown? A methodological consequence of our model is that researchers can identify a visualization's exact role: Which problem representation does it imply and which perspective does it adopt or suggest? Does a visualization merely explicate the information provided by the problem, or does it show the problem's solution? By mapping particular aspects of the Bayesian problem space to specific visual features, future studies of visual aids can measure the interplay between the task's psychological demands, visual features of representations, and viewers' background knowledge and graphical literacy much more precisely.

### 5.2. Perspectives on Bayesian Brain Teasers

Psychology has a long tradition of studying Bayesian problem solving with toy tasks that serve as entertaining brain teasers and appear to show people's inability for straight thinking (e.g., Kahneman and Tversky, 1973; Bar-Hillel, 1980; Bar-Hillel and Falk, 1982). Such tasks let probabilistic events unfold within some narrative and lure most naïve participants into providing an intuitive, but false solution.

To demonstrate the generality of our model, we first use it to explicate another notorious instance of *base rate neglect* (e.g., Kahneman and Tversky, 1973; Tversky and Kahneman, 1974). A famous problem in this area is the *cab problem* (originally introduced by Kahneman and Tversky, 1972a, and extensively analyzed by Bar-Hillel, 1980; Birnbaum, 1983; Macchi, 1995):

A cab was involved in a hit-and-run accident at night. Two cab companies, the Green and the Blue, operate in the city. You are given the following data:

1. 85% of the cabs in the city are Green and 15% are Blue.
2. A witness identified the cab as a Blue cab. The court tested his ability to identify cabs under the appropriate visibility conditions. When presented with a sample of cabs (half of which were Blue and half of which were Green) the witness correctly identified each color in 80% of the cases and erred in 20% of the cases.

What is the probability that the cab involved in the accident was Blue rather than Green?

This problem description provides base-rate information [i.e., the prevalence of both types of cabs: *p*(Green) = 0.85, *p*(Blue) = 0.15], diagnostic information (i.e., the reliability of the witness testimony: *p*(blue|Blue) = *p*(green|Green) = 0.80), and asks for an inverse conditional probability (i.e., *p*(Blue|blue)). The problem's correct solution is 41%, but the median and mode of participants' answers in empirical studies is 80%, thus coinciding with the credibility of the witness and appearing to neglect the base rate information.

**Figure 9:**
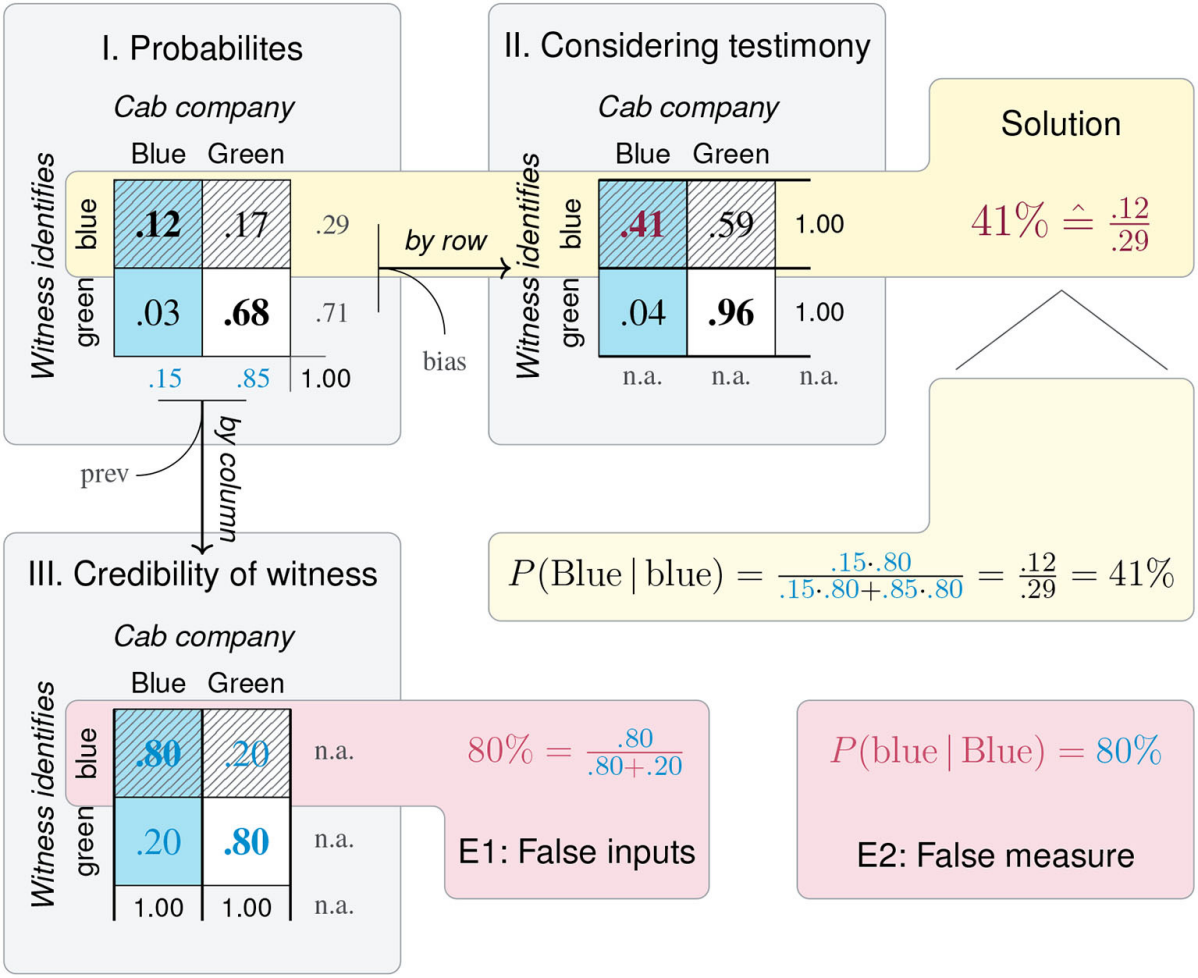
The *cab problem* (Kahneman and Tversky, 1972a) corresponds closely to the *mammography problem* by providing *base rate* information [e.g., the *prevalence* of *p*(Blue) = 0.15] and two types of diagnostic information (*p*(blue|Blue) = *p*(green|Green) = 0.80, indicating the testimony's *sensitivity* and *specificity*). The problem's solution is *p*(Blue|blue) = 0.41, which is the inverse conditional probability of the given *sensitivity* *p*(blue|Blue) = 0.80, and corresponds to the PPV of the *mammography problem*. The analysis explains the problem's difficulty and reveals two ways of erroneously answering 80% (**E1** vs. **E2**) that explicate the informal notions of *base rate neglect* and *representativeness*. Panel **I** contains probabilities, whereas Panels **II** and **III** contain conditional probabilities. (Blue cells mark Blue cabs; shaded cells mark testimonies of “blue”; red areas mark potential errors; yellow areas highlight the solution's perspective.)

The problem information can be used to frame a 2 × 2 matrix that cross-tabulates an actual condition (*Was the cab Blue or Green?*) with two alternative witness testimonies (*Does the witness report a blue or green cab?*). Figure 9 locates the details provided by the problem (shown in blue) in our explanatory framework. This reveals the close correspondence of the *cab problem* to the *mammography problem* (see Figure 4). Again, the provided conditional probabilities (in Figure 4III) adopt a *by column* perspective on an implicit 2 × 2 matrix that can be reconstructed by multiplying each condition's specific information (i.e., the *sensitivity* and *specificity* of the witness) by the corresponding base rates (for Blue vs. Green cabs). Geometrically, solving the problem by Bayes' theorem requires first reversing the implicit *by column* perspective (to compute the joint probabilities of Panel I) and then adopting an orthogonal *by row* perspective (to derive the desired conditional probability *p*(Blue|blue), shown in red, and corresponding to the mammography's PPV).

Interestingly, this analysis reveals two distinct rationales for erroneously answering 80%. First, participants could divide the top-left cell by the row sum, but erroneously use the conditional probabilities (of Figure 4III), rather than the unconditional probabilities (of Figure 4I). This error of *false inputs* (E1) explicates the essence of *base rate neglect* as performing the right calculation with the wrong inputs. A merely informal account of this notion could easily confuse it with another error, which also ignores all base rates. This second error fails to distinguish *p*(Blue|blue) from its inverse *p*(blue|Blue) and reports the testimony's *sensitivity* or *specificity* as the desired answer. Mistakenly reporting a *false measure* (E2) as the solution has been labeled as an *inverse fallacy* (Eddy, 1982; Koehler, 1996) and attributed to using a *Fisherian* algorithm (Gigerenzer and Hoffrage, 1995) or *representative thinking* (Dawes, 1986; Zhu and Gigerenzer, 2006). The prominent hypothesis that a *representativeness heuristic*, which uses similarity or the degree of correspondence of an instance to a category as a proxy for judging its probability, may cause and explain the observed errors (Kahneman and Tversky, 1972b, 1973), has been criticized as overly narrow and vague (Gigerenzer, 1991, 1996). As accounts of *representativeness* typically invoke notions of saliency and correspondence, they can be consolidated with our structural attempt for rendering task representations and problem solutions more obvious. The fact that our model is much narrower than an arguably vague notion may actually be a benefit: Not only does it allow us to pin-point the precise location of potential errors, but also offers a new role for *representativeness* as explaining *why* people preferentially adopt the mis-leading *by column* perspective.

Our framework can accommodate problems that feature more than two options. For instance, the *three-door* or *Monty Hall problem* (Selvin, 1975; vos Savant, 1990) is named after a TV show in which a contestant faces a choice between three doors (*D*_1_–*D*_3_). Behind one random door lurks the grand price of a car, whereas each of the other two doors conceals a goat. After the contestant selects a door (e.g., *D*_1_), the host (who knows all objects' locations) opens another door (e.g., *D*_3_) to reveal a goat. The question whether the contestant should now switch to the other door (*D*_2_) has sparked an intense public debate and inspired extensive studies (e.g., Granberg and Brown, 1995; Krauss and Wang, 2003; Baratgin, 2009).

**Figure 10:**
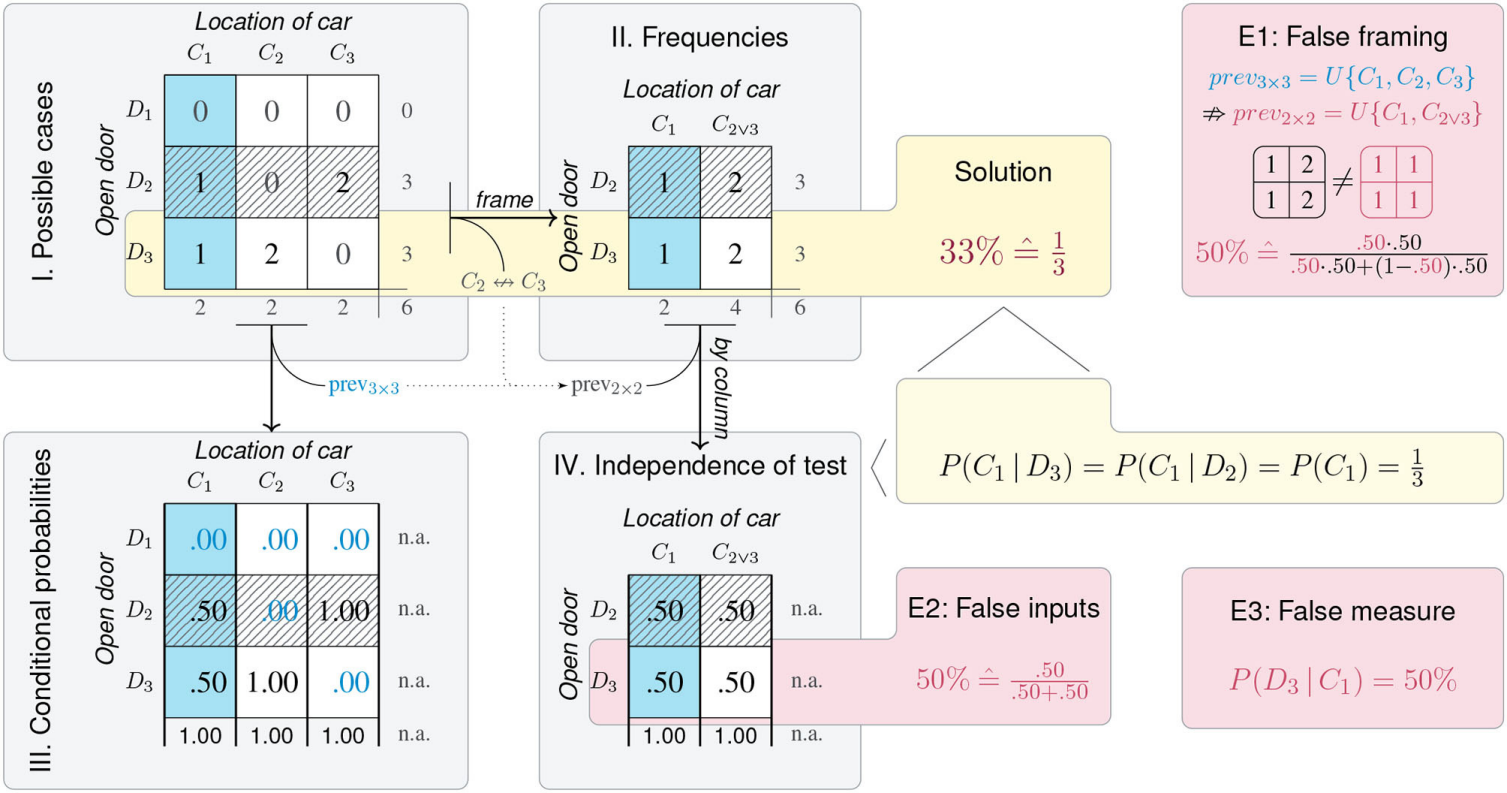
Explicating the *Monty Hall problem* (vos Savant, 1990) in its standard two-door scenario (Krauss and Wang, 2003). The 3 ×3 matrices map three equiprobable car locations (*C*_1_–*C*_3_) to the three doors that the host can open (*D*_1_–*D*_3_) and depict all possible combinations after the contestant selects *D*_1_ in terms of frequencies **(I)** and conditional probabilities **(III)**. Removing the distinction between *C*_2_ and *C*_3_ and the impossible row *D*_1_ frames a 2 × 2 matrix that illustrates the contestant's dilemma **(II)**. Adopting a *by row* perspective yields the solution *p*(*C*_1_|*D*_3_)$=\frac{1}{3}$ and *p*(*C*_2∨3_|*D*_3_)$=\frac{2}{3}$, indicating that the contestant should switch doors. Adopting a *by column* perspective on the same matrix yields *p*(*D*_*i*_|*C*_*j*_) =.50 for all combinations **(IV)**, indicating that conditionalizing the host's action on the car's location is uninformative. The fact that three potential errors (i.e., *false framing*, **E1**, *false inputs*, **E2**, and reporting a *false measure*, **E3**) all yield the same erroneous value of 50% explains why this false intuition is so compelling. (Blue cells mark the contestant's initial choice; red areas mark potential errors; yellow areas highlight the solution's perspective.)

Explicating the *Monty Hall problem* by our model extends the previous examples in two ways: First, accounting for a probabilistic task with three options renders the mapping from narrative to diagnostic scenario more challenging. Second, the standard two-door scenario of the problem (in which the host reveals a goat and the contestant thus seems to face a choice between two remaining doors, Krauss and Wang, 2003) departs from the problems discussed so far by requiring that the interplay between the situation and the host's options must be taken into account. Figure 10 depicts the standard scenario as a 3 ×3 matrix (on the left): Its *X*-dimension denotes the three possible locations of the car (*C*_1_–*C*_3_) and its *Y*-dimension denotes the three doors that the host can open (*D*_1_–*D*_3_). Figure 10I indicates the number of possible cases as the host's options for opening doors given the contestant's initial choice and the car's actual location. As there are *N* = 3! = 6 possible arrangements of a car and two distinct goats and each car location is equiprobable (i.e., *U*{*C*_1_, *C*_2_, *C*_3_}), each column contains two cases. If the contestant initially selects *D*_1_, only *D*_2_ or *D*_3_ can be opened. Which of these doors *is* opened depends mostly on the car's location: If the car is at *C*_2_ or *C*_3_, the host must open *D*_3_ or *D*_2_, respectively, to reveal a goat. If the car is at *C*_1_, both *D*_2_ or *D*_3_ hide goats and could be opened, but we assume that the host has no preference and hence opens both doors equally often in those cases. The lower 3 ×3 matrix (Figure 10III) expresses the same setup in terms of probabilities that are conditionalized on car location (i.e., *by column*). Whereas, only the host can know which of the four possible combinations (i.e., non-zero cells in Figures 10I,III) is realized in an actual game, a savvy contestant could reconstruct all possible cases and their probabilities from the problem description. But even if an appropriate matrix is framed, a crucial element for solving the problem consists in adopting the right perspective on it.

To further clarify the contestant's dilemma, we frame the initial 3 ×3 matrix as a 2 × 2 matrix that collapses *C*_2_ and *C*_3_ into one column (to only distinguish *C*_1_ from *C*_2∨3_) and removes the impossible row *D*_1_ (Figure 10II). As in our previous examples, we can now adopt a *by row* or a *by column* perspective on this matrix. The problem's solution is derived by conditionalizing *C*_1_ on the identity of the opened door (i.e., *by row*). Using either a 3 ×3 or the 2 × 2 matrix (Figures 10I–III), this shows that $p\left(C_{1}|D_{3}\right)=p\left(C_{1}|D_{2}\right)=\frac{1}{3}$. Thus, the conditional probability that the car is at *C*_1_ given that either *D*_3_ or *D*_2_ has been opened is identical to its original probability $p\left(C_{1}\right)=\frac{1}{3}$. By contrast, adopting the same perspective on any alternative door shows that $p\left(C_{2\vee 3}|D_{2}\right)=p\left(C_{2\vee 3}|D_{3}\right)=\frac{2}{3}$, implying that the contestant should switch in both cases.

Although switching doors would double the contestant's chances for winning the car, 87% of naïve participants prefer to stick with their initial choice (Granberg and Brown, 1995). A key argument for their inertia is the intuition that the host's elimination of a losing option creates a new situation that implies a 50–50 chance of winning with each of the remaining doors. This *uniformity* belief (Falk, 1992, p. 202) ignores that the host's action depends on both the contestant's choice and the car's location and falsely assumes that the game is re-set after a goat has been revealed (see Baratgin, 2009, for an analysis of this *updating* interpretation). In our model, the false assumption of two equiprobable options (i.e., *U*{*C*_1_, *C*_2∨3_}) would frame an erroneous 2 × 2 matrix in which all cell values were equal. As such a matrix would fail to reflect the actual situation, we refer to this error as *false framing* (E1). Once such a misleading 2 × 2 matrix has been framed, the illusion that the chance of winning is 50% for either option is inevitable, as it would follow from adopting any arbitrary perspective on it.

Interestingly, our analysis shows two additional options for the same conclusion. Adopting a *by column* perspective on the correct 2 × 2 matrix (Figure 10II) yields a 2 × 2 matrix that contains values of 0.50 in all of its cells *p*(*D*_*i*_|*C*_*j*_) (Figure 10IV). This essentially means that the door opened by the host is an uninformative diagnostic test when conditionalizing on the car's location (*by column*), rather than on the identity of the open door (*by row*). Assuming this unhelpful perspective on a correct 2 × 2 matrix, the error of *false inputs* (E2) would perform the right calculation on the wrong inputs and constitute another instance of *base rate neglect*. Similarly, computing the inverse of the actually relevant conditional probability [i.e., *p*(*D*_3_|*C*_1_), rather than *p*(*C*_1_|*D*_3_)] would report a *false measure* (E3) and could be described as an *inverse fallacy* or resulting from a *Fisherian* algorithm or *representative thinking* (see above). However, the fact that *all* of these errors yield the same value of 50% may explain why this false intuition is so compelling.

Having explicated three notorious problems of Bayesian reasoning by our framework, we trust that analogous accounts could illuminate related problems—like the *engineer-lawyer problem* (Kahneman and Tversky, 1973), the *conjunction fallacy* (Tversky and Kahneman, 1983), or the *three-prisoners problem* (Falk, 1992)—and more remote phenomena, like the *class-inclusion task* (Politzer, 2016), or *Simpson's paradox* (Simpson, 1951). Our model explains their difficulty by the interplay of two factors: (a) the challenge of constructing an appropriate problem representation, and (b) a discrepancy between an implicit perspective adopted by the problem information and the perspective required for the solution. The first obstacle lies in framing an appropriate 2 × 2 matrix. This is particularly challenging when the problem involves three or more options that obscure the binary nature of the underlying diagnostic test. But even if an appropriate 2 × 2 matrix has been framed, the specific information provided by the problem can still be misinterpreted or may shift the reasoner's focus into a misleading direction. A purely analytic account can reveal and distinguish between potential errors, but not disentangle them any further. While adopting the right perspective on an appropriate representation may also make a problem's solution transparent, our model's main purpose consists in explicating problems structures and pinpointing potential errors, rather than resolving them.

Despite their theoretical appeal and practical ramifications, textbook problems of Bayesian reasoning require only a small part of our overall framework. In fact, the scope of the matrix lens model also extends beyond the domain of classification and clinical diagnostics that comprise the majority of measures defined in Table 3. To illustrate its generality, we now address a pertinent question raised in our introductory example.

### 5.3. Perspectives on Surviving the Titanic

When using the population of *Titanic* passengers to illustrate the initial steps of our model (in sections 2.1, 2.2), we evaded the most obvious question: Who survived the disaster? A more nuanced version of this query would aim to identify factors that contribute to a passenger's survival. Given that an emergency protocol known as the *Birkenhead drill* demands the preferential rescue of women and children when abandoning a ship, a seemingly straightforward question would ask: Were women and children successfully rescued first?

Before addressing this question, we need to prohibit two simplistic answers. For instance, a categorical interpretation of the drill would require that *all* women and children must be saved prior to rescuing any adult male. However, given that the disaster killed over two thirds of the ship's population (67.7%, see Figure 3), demanding that the victims must not contain a single female or child seems overly conservative. Similarly, adopting a continuous approach but merely counting the victims or survivors per group would ignore their base rates, which are heavily skewed toward adults and males. Rather than comparing the frequencies of individual cells, our model should enable us to derive a comprehensive measure that provides a quantitative answer to the question: To *what degree* was the policy implemented? Interestingly, this is surprisingly difficult and implies making several choices that substantially shape our answer.

**Figure 11:**
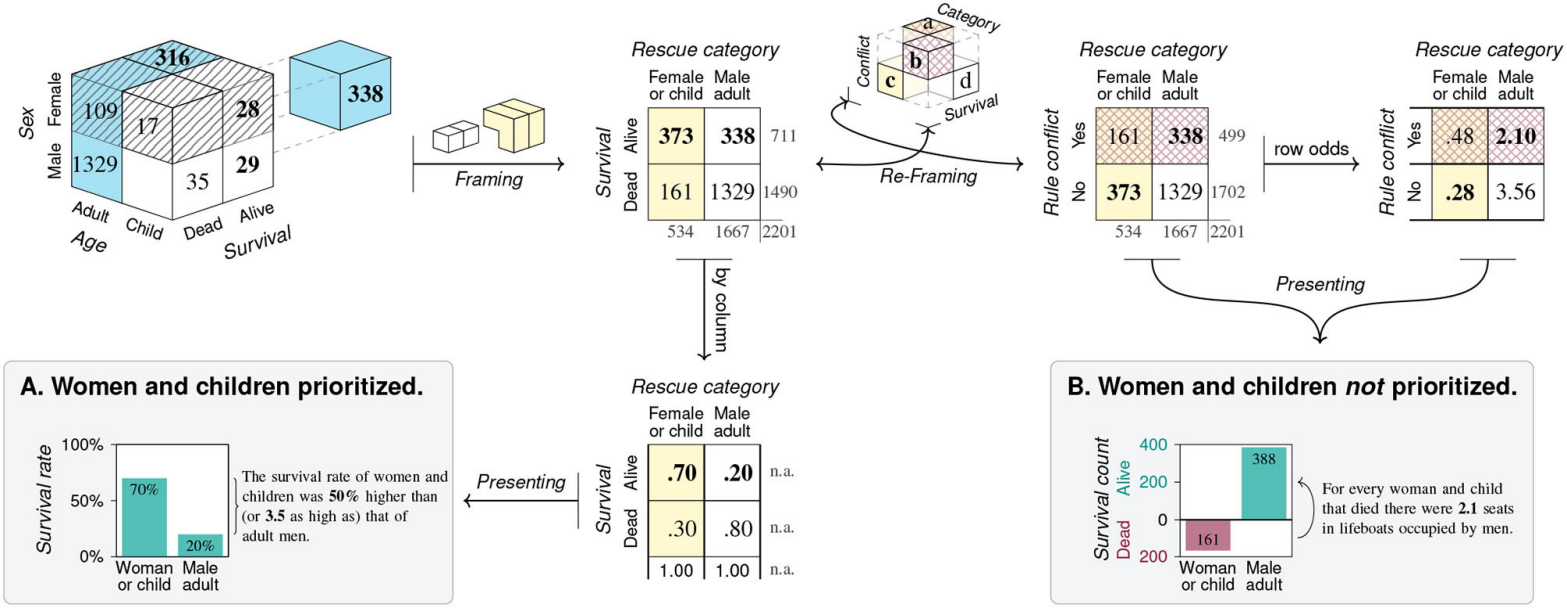
Applying the matrix lens model to evaluate whether women and children on the *Titanic* were successfully rescued first. Any answer depends on the policy's interpretation and the perspectives adopted on the data. Comparing survival rates between groups suggests that women and children received preferential treatment, but computing row odds for cases in conflict with the policy supports the opposite conclusion. Presenting only one measure in a non-transparent fashion (as in **A** vs. **B**) would obfuscate the problem, rather than solving it.

Our analysis assumes a binary grid of the *Titanic*'s population (see section 2.1) and begins by framing an appropriate 2 × 2 matrix (section 2.2). Although Figures 3A–C provide three alternative perspectives on the three-dimensional *Titanic* data, none of them allows answering our question. For rather than expressing *Survival* as a function of *Age* (Figure 3B) or *Sex* (Figure 3C), measuring the drill's success requires a 2 × 2 matrix that collapses female adults and children of both sexes into a combined *Rescue category* and contrasts their *Survival* status with that of male adults. This matrix can be constructed from the binary grid and is shown in Figure 11. Evaluating this matrix is a matter of perspective: For an individual of either group, being *Alive* is certainly better than being *Dead*. However, viewing the 2 × 2 matrix from the drill's normative angle implies that saving a female or child is preferable to saving a male adult. If there are victims among female and children, any adult male survivor may face misgivings. Due to this constellation, the diagonal of the 2 × 2 matrix does not denote *accuracy*, but rather whether a category combination can or cannot conflict with the policy. Our model's crucial step of *focusing* (section 2.3) adopts a particular perspective on the 2 × 2 matrix to derive a measure that captures the desired aspect. To illustrate that this step includes important choices, we adopt two distinct perspectives:

1. *Comparing survival rates*: To control for the base rates of both *Rescue categories*, we adopt a *by column* perspective on the 2 × 2 matrix and compute each group's chances of survival (see the measures of *absolute risk*, AR, in Table 3). This reveals that the survival rate of male adults was only 20%, whereas the survival rate among women and children was 70% (or mortality risks of 80 and 30%, respectively). The difference between both risks can be expressed as an *absolute risk reduction* (ARR) of 50% for women and children or—possibly inflating the effect—as an increase of the *relative survival rate* of women and children by a factor of 2.5 (relative to adult males). As relative risks are notoriously misleading (Gigerenzer et al., 2007), simply contrasting the absolute magnitude of both survival rates suggests that women and children were prioritized.
2. *Computing odds for conflict cases*: An alternative perspective on the same matrix directly contrasts the cells that can conflict with the rescue policy. Re-framing the matrix arranges it so that its former diagonals form its rows. Focusing exclusively on the top row contrasts 161 women and children who died with 338 adult men who survived. Importantly, the larger number of the latter group implies that there was sufficient rescue capacity for saving *all* women and children. Computing the *odds* between both numbers reveals that for any dead woman or child there were 2.1 seats in lifeboats occupied by adult men. Although the magnitude of this value seems similar to the relative risk factor of 2.5 (in 1), it points in the *opposite* direction and suggests that women and children were *not* prioritized.

Obtaining two results with opposite conclusions presents us with a puzzle: Which answer is correct? Actually, as either result is incomplete, rather than wrong, both results together allow for a more balanced assessment of the rule's success: While women and children survived at a considerably higher rate than male adults, a better allocation of seats in lifeboats would have boosted their survival chances even further. Interestingly, each individual result could easily be mistaken as the only one and be used to mislead people. By accurately reflecting a particular aspect of the problem, each result obscures the original information and prevents an alternative perspective. Especially when only communicating the value of some cryptic measure and showing a seemingly informative, but decidedly non-transparent visualization (see Figures 11A,B), the manipulative potential of any such analysis is substantial.

The lesson to be learned here is *not* to stop analyzing data or to avoid drawing conclusions. Instead, we must learn to be skeptical about seemingly objective measures that remain non-transparent. As we have shown, adopting perspectives is an inevitable part of the scientific process and the price to be paid for the benefits of abstraction and specialization that come with particular measures. Thus, the antidotes to ignorance and pseudo-scientific propaganda are not doubts or disdain for highly-specialized scientific tools, but their profound comprehension and transparent communication within a risk-savvy society (Gigerenzer and Gray, 2011; Gigerenzer, 2014). Dealing flexibly and responsibly with alternative perspectives and results requires a level of insight into the meaning and limits of measures that goes beyond mere rote learning of definitions and formulas. While our theoretical model may contribute to a better understanding of metrics and their proper interpretation, the key challenge for educators and instructors is to design effective training programs that render scientific insights more transparent for scientists, their audiences, and students (Martignon and Hoffrage, 2019).

## 6. Discussion

In this article, we link the basic construct of a 2 × 2 matrix to the typical semantic interpretations of binary dimensions that are of interest in different domains. This explains a large variety of scientific measures in a unifying framework. We illustrate how our model can be applied to explicate notorious problems of Bayesian reasoning, as well as to address scientific questions of a more general nature. While this highlights the problems' structural similarities and pinpoints potential errors more precisely than previous explanations, it also reveals that the selective and organizational processes of *filtering, framing*, and *focusing* imply characteristic trade-offs: The price of increasing resolution on some particular aspect is a loss of detail and context. Importantly, any perspective adopted in the derivation of a measure is rendered implicit and encapsulated in its numeric value. Thus, a transparent communication and visualization of scientific results needs to explicate the perspective adopted in their derivation.

Although we trust that our approach makes contributions to various fields, some caveats may help to pre-empt possible misunderstandings. Rather than providing a unique account. our model stands in a long tradition of expressing cognitive phenomena in visual metaphors (see Supplement 2). Regarding our goals, we provide an analytic tool for studying problems, not a recipe for resolving them. Although our model is abstract and flexible enough to be applied to other problems, its structural mapping to a specific problem is not always straightforward. Thus, our approach may help others in solving similar problems, but such benefits are not automatic and yet to be shown. Similarly, this article uses visualizations to render our model's steps and processes more concrete (see Figures 2–5), but the model itself is abstract, rather than visual in nature. Whereas, most steps of our model (i.e., the steps of *filtering, framing*, and *focusing*) are descriptive, its final step (*presenting*) allows for prescriptive applications. But even when using our notion of *transparency* for evaluating visualizations of numeric measures, there is no guarantee that those that conform to our definition will yield benefits in comprehension or performance. Thus, our model can be used to generate hypotheses, but their success and reach remains to be tested in empirical studies.

Overall, analyzing tasks in the form and terms of 2 × 2 matrices is primarily a methodological tool for revealing structural similarities between problems and suggests where to look for possible errors and solutions. By contrast, our framework is silent about which perspective solves a given problem, nor provides us with a magic potion that adopts the right perspective on all problems. As all models are wrong on some level, ours must prove its worth by changing our reader's perspectives on related problems.

## 7. Conclusion

Could you restate the problem?

Could you restate it still differently?

(Polya, 1957, p. 75)

In the 1999 science fiction movie *The Matrix*, swallowing a red pill reveals the world as a technological projection: Everything perceived to be real turns out to be a mere illusion. Real science is less spectacular, but also full of projections. And in sharp contrast to the action thriller, adopting particular perspectives is in fact a theoretical tool for gaining insights and discovering meaningful relations about the world.

The matrix lens model illustrates a sequence of steps that filter information, frame it as a 2 × 2 matrix, and focus on increasingly specific aspects of the world. Adopting distinct perspectives on the shared structural construct of the 2 × 2 matrix yields a rich variety of measures that enable high levels of abstraction and specialization. But any gain in the resolution of details comes at the cost of reducing generality and limiting the scope of possible conclusions. Beyond explicating the dialectic epistemology of scientific measures, the model integrates a rich variety of concepts into a common framework. Our geometric approach shows the shared underlying structure of many semantic domains, highlights links between a confusing range of measures, and may help to clarify or resolve several academic debates.

Applying our model to both theoretical and practical problems provides new perspectives on them. From a theoretical stance, our model suggests structural explanations for the well-known facilitation effects of frequency formats, and precisely describes potential errors in related problems of Bayesian reasoning. By explicating the representational nature of such problems, we show how a shift in perspective essentially solves them. With regard to solving scientific problems by analyzing data, our model reveals the choices inherent in the selection of measures and cautions against drawing premature conclusions on the basis of seemingly objective values. As any quantitative measure selectively illuminates some aspect of the world and encapsulates the perspective adopted in its derivation, we should be skeptical whenever facing results that we do not fully understand or are not presented in a transparent fashion.

Visual illusions do not disappear by explaining them. But once we become aware that an ambiguous image can alternatively be seen as a rabbit or a duck, our familiarity with the image can ease the flip between both interpretations. Consequently, it should not surprise us that representational problems persist even when their underlying mechanisms become transparent. For students of clinical diagnostics, it will remain perplexing that medical tests with high sensitivity and specificity can still exhibit poor predictive values. Similarly, it will continue to seem peculiar and vexing when two measures that adopt different angles on the same data support opposite conclusions. But realizing that such phenomena are neither paradoxical nor inconsistent is an intellectual step that requires instruction and training. Thus, understanding that conflicts between measures—or between people reporting them as facts—are an inevitable consequence of their inherent perspectives is an important insight on the path to scientific literacy.

The red pill to swallow for the scientific enlightenment of modern societies lies in translating these insights into an educational strategy. Given the key role of perspectives for the meaning and interpretation of scientific measures, understanding how measures encapsulate particular viewpoints is an important skill for scientists and their audiences. The costs incurred by this explication are outweighed by the fact that scientists stand to benefit twice from embracing the representational nature of their investigations: Beyond enabling them to choose their measures more responsibly and wisely, a more transparent communication of their results may also enable more trust in their findings.

The notion of *insight* implies suddenly seeing a solution. As we have shown, adopting the right perspective on a problem makes its solution obvious—it becomes simple and transparent. We show that capturing scientific measures and explicating problems in terms of adopting particular perspectives on the structural construct of a 2 × 2 matrix reveals aspects that remain obscure in any isolated treatment. We trust that readers will discover additional opportunities for framing problems in this form and hope that viewing them through the lens of a 2 × 2 matrix will render their solutions more transparent.

## Author Contributions

HN, NG, and DS contributed to the conceptual development of the work. HN wrote the first draft of the manuscript. NG and DS provided substantial revisions. DK and WG provided general guidance and critical revisions. All authors contributed to the article and approved the submitted version.

## Conflict of Interest

The authors declare that the research was conducted in the absence of any commercial or financial relationships that could be construed as a potential conflict of interest.

## Footnotes

^1^While the non-reversible nature of conditional probabilities seems puzzling in the abstract, an example makes it obvious: Given the population of all U.S. citizens from 1789 to 2020, the conditional probability *P*(male|U.S. president) = 1, but the inverse conditional probability *P*(U.S. president|male) is almost zero.

^2^DOR is a quadrangular score (see its definition in Figure 6IV) that can also be calculated by first adopting a *by row* perspective on the matrix, computing two column-wise likelihood ratios, and then their odds ratio. Thus, DOR values are also independent of bias.

^3^In technical terms, providing *p*(*T*) and *p*(*C* ∩ *T*)—or the corresponding joint frequencies—no longer requires Bayes' theorem for computing the posterior probability *p*(*C*|*T*) from a prior *p*(*C*) and the likelihoods *p*(*T*|*C*) and *p*(*T*|*eg C*).
