# Supplementary material for: Perspectives on the 2 × 2 Matrix: Solving Semantically Distinct Problems Based on a Shared Structure of Binary Contingencies
Source: Front Psychol. 2021 Feb 9;11:567817. doi: 10.3389/fpsyg.2020.567817 (PMC7901600; doi:10.3389/fpsyg.2020.567817)
Supplement: Supplementary file 1 [file Presentation_1.pdf]

Supplementary Material

The following sections provide additional information on specific aspects. Part 1 provides technical details on some claims made in the article, whereas Part 2 adds an historical perspective.

1 AN INVENTORY OF MATRIX PROJECTIONS

Our article emphasized that 2×2 matrices are compact, yet flexible representational constructs. This claim partly rests on the fact that any 2×2 matrix can be framed in $2^3 = 8$ spatial variants (see Figure S1). Despite their differences in surface structures, all matrices that result from mirroring rows and columns or rotating a matrix to swap its dimensions express identical semantics. A lack of conventions for framing 2×2 matrices contributes to their confusing nature. For instance, when distinguishing between type-I and type-II errors (α vs. β), there is no agreement between authors of introductory statistics texts whether to depict the true state of the world as rows (Bortz and Schuster, 2010; Kempf, 2006; Leonhart and Lichtenberg, 2009) or as columns (Howell, 2013; Statistical hypothesis testing, 2020) of a 2×2 matrix.

By contrast to these trivial variants, the three matrices resulting from projecting a three-dimensional cube (with dimensions X, Y, and Z) along one of its dimensions frame semantically different 2×2 matrices (see Figures 3 and 5). Interestingly, the surface changes of the 2×2 matrix variants requiring different interpretations are smaller than those of the trivial variants: They only swap two cells of a single row or column, rather than swapping or rotating entire rows or columns. The key contribution of our model consists in explicating how these matrices can accommodate different task domains and that adopting particular

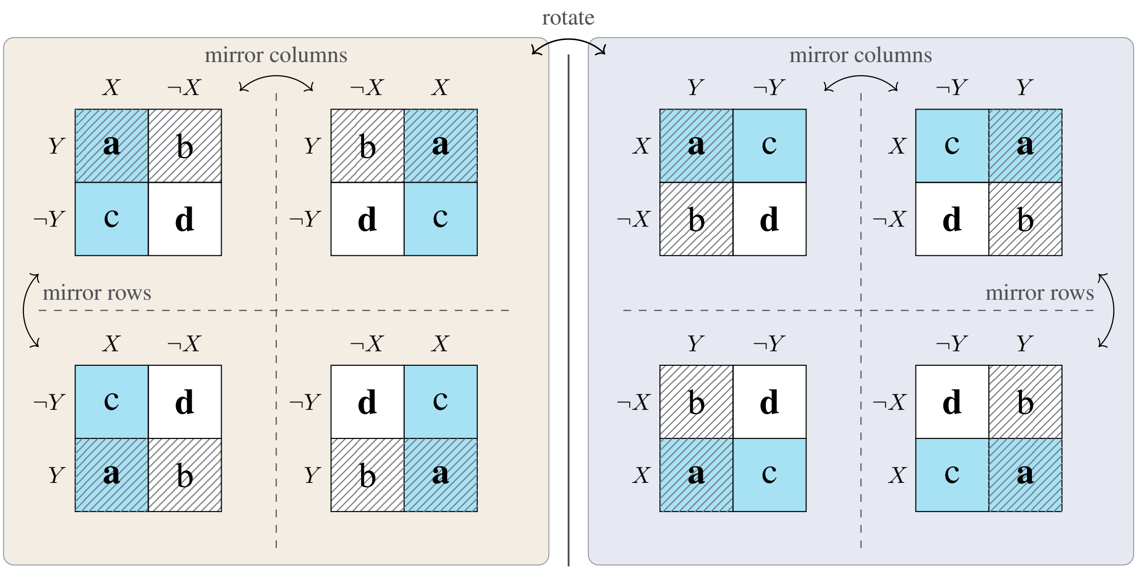

Figure S1. Spatial variants of a single 2×2 matrix. Mirroring rows or columns and rotating dimensions allows framing $2^3 = 8$ variants of the same matrix. Despite different surface structures, all these matrices express identical semantics. Conceptually, the matrices within a quartet (i.e., on the left vs. right of the vertical line) differ by their *assignments* (i.e., the locations of binary category levels), whereas those between quartets differ by their *layouts* (i.e., the order of the dimensions X and Y, see Table S1). Cell background color marks category X; pattern marks category Y; bold fonts mark category correspondence.

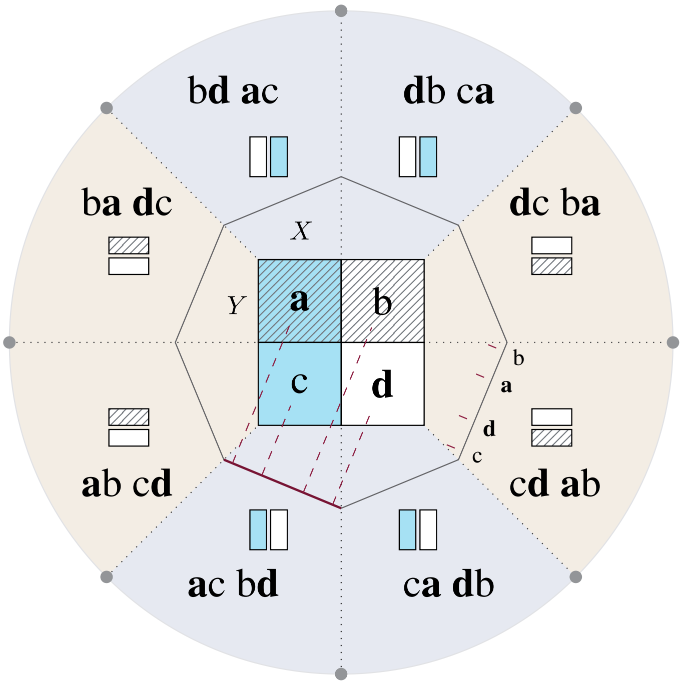

Figure S2. All linear projections of a 2×2 matrix into a list of matrix cells (from 2D into 1D). Each list results from projecting the matrix cells on a line that discriminates between all four cells (and parsing them in counter-clockwise fashion). The eight different lists form two types of hierarchical trees that adopt either a *by column* perspective (X, Y) or a *by row* perspective (Y, X) on the matrix. Each type of tree exists in four trivial variants that swaps the order of its leaves. Dotted lines mark orientations in which at least two cells are indistinguishable in the one-dimensional projection.

perspectives on a shared underlying structure gives rise to a wide variety of measures and representational effects.

It has previously been noted that a 2×2 matrix contains two hierarchical trees (e.g. Kurz-Milcke et al., 2008, p. 20), which has been exploited by visualizing double trees (Wassner et al., 2004) and frequency nets (Binder et al., 2020). These analyses recognize the special status of the 2×2 matrix framed by X and Y and adopt a *by row* and *by column* perspective on it (see Figure 5A), but provide an incomplete account of the underlying task structure. In Sections 2 and 3, we show that the actual semantics of the task domain are three-dimensional. Its 2D-projection on a 2×2 matrix $\{X, Y\}$ implicitly represents a third dimension Z as its diagonal that — if explicated — gives rise to two additional matrices (see Figures 5B and 5C). Further projecting these 2×2 matrices into linear representations yields multiple lists (or quadruples of cells) per matrix. Each list can be augmented into an hierarchical tree that imposes an order on the explicit matrix dimensions.

Given that each 2×2 matrix actually contains three dimensions, we can ask: How many distinct lists and trees does a given matrix support? The answer depends on the flexibility of the rules that parse the four matrix cells. If we restrict these rules to linear projections (i.e., reading out the sequence of cells as they are projected on a line that assumes any angle that allows distinguishing between all four cells), each matrix supports eight distinct lists. Figure S2 shows these linear projections of a 2×2 matrix into a list of four matrix cells (from 2D into 1D). As the eight resulting lists never contain both cells of a matrix diagonal (e.g., a and d) in the same pair, they correspond to the eight trivial spatial variants of a 2×2 matrix (shown

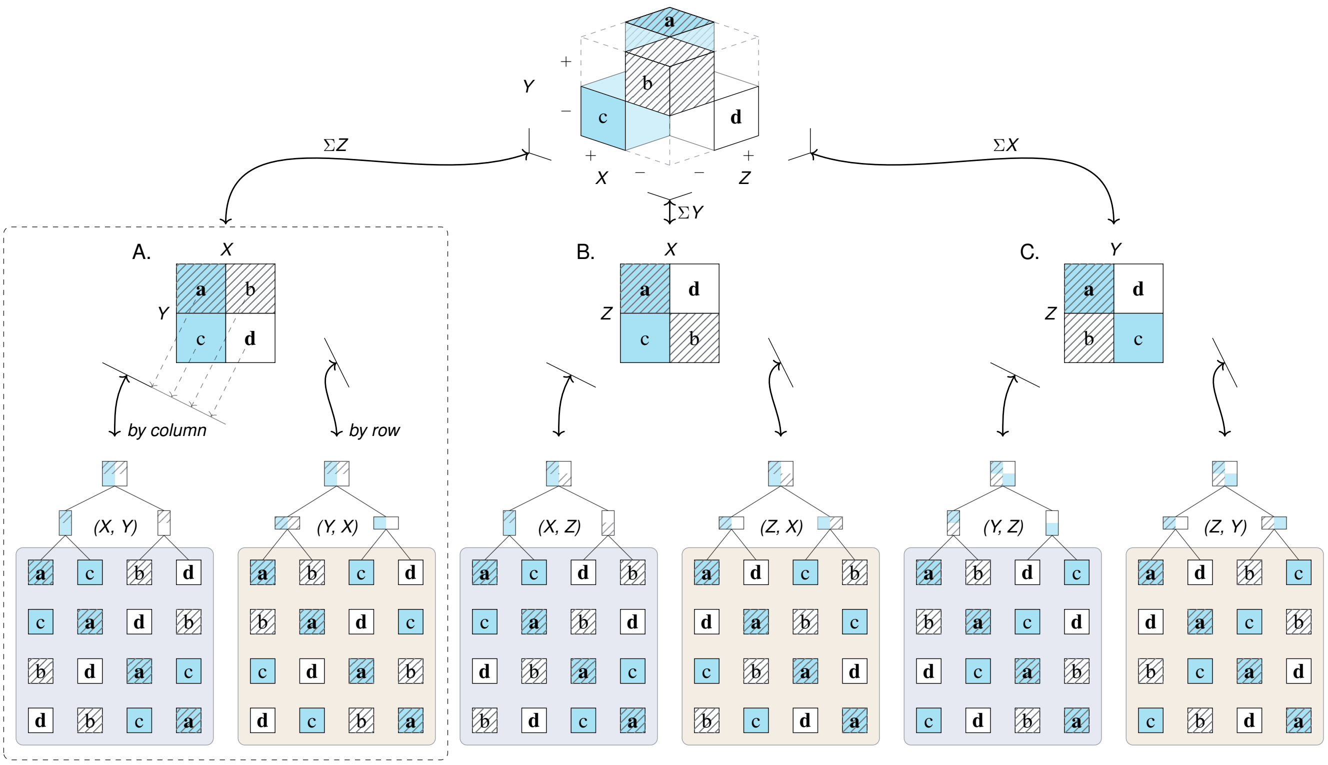

Figure S3. The partial cube model showing all 24 distinct variants on its 1D-level. Importantly, the same 24 variants are available on each representational level. For the 3D-model, the 24 perspectives result from viewing each of 6 sides of the cube in 4 orientations. For the 3 distinct 2D-models, each 2×2 matrix exists in 8 trivial variants. For the 6 distinct 1D-models, each list or tree can be arranged in 4 trivial variants. While all perspectives are informationally equivalent, the dashed region marks the 2D- and 1D-visualizations that are semantically privileged for tasks in which the correspondence dimension Z can remain implicit. Cell color marks category X; pattern marks category Y; bold font marks correct classifications Z.

in Figure S1). Thus, when classifying the eight lists by pairs, they form two categories that either adopt a *by column* perspective (X, Y) or a *by row* perspective (Y, X) on the matrix. Thus, each 2×2 matrix gives rise to exactly two distinct hierarchical trees. The four lists describing the same tree show its $2^2 = 4$ trivial variants resulting from swapping the direction of its branches at both levels. Figure S3 incorporates these insights into Figure 5 and shows the partial cube model with all 24 possible variants on the 1D-level.

A strong representational claim of our model is that any task that conforms to the semantics explicated in Section 3 gives rise to the same variants on different levels of visualization. Specifically, the original 3D-structure of the task domain is the generative toolbox that underlies all other representations. By adopting particular perspectives on this structure, it can be viewed as three distinct 2×2 matrices (in 2D) and six distinct lists or trees (in 1D). The representations on all three levels are informationally equivalent and each level is both *sufficient* and *complete* in the sense of providing the full and exhaustive set of all possible 2D- and 1D-projections of the 3D-structure. Showing the informational equivalence of all representations (in the sense of Larkin and Simon, 1987) is straightforward: Any quadruple of cell values (a, b, c, d) can simply be rearranged as a cube, matrix, or list. We now have all puzzle pieces to show the sufficiency and completeness on each level: Figure S3 illustrates the correspondence of the 3D-model to three 2×2 matrices (in 2D) and 24-lists (in 1D). Each of the three 2×2 matrices has eight trivial variants (see Figure S1) and corresponds to two types of of trees (see Figure S2). Thus, the 24 distinct lists

Model Dimensionality	D	Perspectives: Assignments	Frames	Layouts	Total	d	Definition	Choices
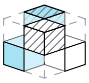	3	8	—	—	24	3	$\{X, Y, Z\}$	choose frame
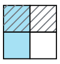	2	4	3	—	24	2	$\{X, Y\}$	choose layout
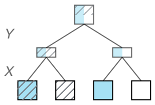	1	4	3	2	24	2	(Y, X)	
**Number of options**	D	2^d	$\binom{3}{d}$	$d!$	24			

Table S1. Enumerating all representational isomorphs of the three-, two-, and one-dimensional models. In three-dimensional space, the three-dimensional nature of the problem can be represented faithfully, as the three model dimensions can independently be assigned to three spatial dimensions ($D = d = 3$, with D indicating model dimensions, and d indicating explicitly represented dimensions). Projecting the original structure to a two-dimensional matrix renders dimension Z implicit ($D = d = 2$), as Z is defined by X and Y. By being aware of this definition (i.e., explicating the perspective), the original problem structure can be restored. Projecting to a one-dimensional list by stacking two dimensions into one ($D = 1$, $d = 2$) further distorts the original problem by imposing an order on the dimensions that is absent from the higher-dimensional models. A hierarchical tree structure explicates this perspective.

correspond to 6 distinct hierarchical trees and 3 distinct 2×2 matrices, all of which are perspectives of a single underlying 3D-structure.

As the same set of perspectives is available on each level, the levels differ not only by the number of model dimensions, but also by the assumed degree of representational stability vs. flexibility. The mechanism of *adopting perspectives* seems to suggest an active adjustment on part of the viewer, while the representation that is being perceived remains passive and inflexible. But as *perspective* is a relational term, this distribution of a dynamic and a static role is arbitrary. A more comprehensive portray of the relation between an object and its mental representation would show a continuum of trade-offs between inertia and flexibility. At one extreme, assuming a completely rigid representation requires that a maximally flexible viewer adopts 24 distinct perspectives for viewing all possible aspects. At the other extreme, assuming a completely inflexible viewer (e.g., someone always parsing cells from top-left to bottom-right) requires 24 different variants of the underlying representation to ensure that each possible aspect is perceived.

Whereas Figure S3 illustrates the equivalence of the 3D-, 2D-, and 1D-models (i.e., partial cube, 2×2 matrix, and list or tree) in a geometric fashion, Table S1 provides a systematic overview by enumerating the possible perspectives on each model dimension. This also shows that each level of visualization enables $4! = 24$ distinct projections. The number of projections on each level is the product of model dimensions D, ways of assigning binary categories (i.e., allocating binary category levels for each explicit dimension d), ways of framing dimensions (i.e., choosing two explicit dimensions out of X, Y, and Z), and selecting one of two possible layouts (i.e., the order of dimensions in lists or hierarchical trees). Table S1 also shows the choices required when designing visualizations with $d = 2$ explicit dimensions: Depicting the original 3D-model $\{X, Y, Z\}$ as a 2×2 matrix requires selecting two explicit dimensions for framing a matrix $\{X, Y\}$ (i.e., rendering one dimension implicit). Further reducing this matrix to a list or tree requires that its dimensions are ordered either as (X, Y), or as (Y, X). Thus, each particular

visualization below the original 3D-structure implies some specialization and imposes representational constraints.

Overall, the 24 perspectives on the 3D-model are all representational isomorphs of each other and provide the complete set of ways in which the 2D- and 1D-visualizations considered here (which only vary by view composition) can depict the underlying situation. Importantly, the informational equivalence of representations leaves ample room for differences in computational efficiency, salience, and semantic interpretations, when being perceived by human viewers. As each visualization type adds distinct visual features (e.g., highlighting less or more salient locations, rendering dimensions explicit vs. implicit, or imposing spatial orders that conform to or conflict with semantic structures), different perspectives of the same underlying structure are perceived differently by human observers, giving rise to a variety of representational effects. Thus, any quest for the best representation boils down to an optimization problem that aims to find a combination that — given the particular constraints of task, representation, and viewer — allows solving the task with a maximum of accuracy or minimum of effort.

2 OPTICAL METAPHORS FOR MENTAL REPRESENTATIONS

Psychology has an established tradition of describing mental representations as if they were external, spatial, and visual, and could thus be examined, observed, and inspected. Although this analogy can be traced back to early philosophers (e.g., Plato's allegory of the cave), perhaps the most striking example of this tendency is the notion of *insight* as the discovery of a problem's solution (see, e.g., Köhler, 1925; Wertheimer, 1959). As others have provided excellent reviews on this topic (see, e.g., Shepard, 2001, and commentaries), we only point out some references that are pertinent in the context of the current article.

The gist and terminology of our model stands in a long tradition of expressing phenomena of human reasoning in visual metaphors. At least since De Finetti's (1937) pioneering study on *foresight* (*prévision*), mechanisms of statistic inference have often been couched in optical terms. Perhaps most prominently, our model's name is reminiscent of Brunswik's eminent *lens model* (Brunswik, 1952). Both models share not only the optical metaphor, but also a premise that insight is mediated — and often distorted — by selective intermediate processes. However, Brunswik's much more ambitious model provides a probabilistic account of perception that views the world through a lens of distal and proximal cues (see Hammond and Stewart, 2001). By contrast, our model suggests an analytic framework for explicating a variety of problems and scientific measures by viewing them through the lens of a 2×2 matrix. Other prominent inspirations for our model include comparisons of heuristics and biases to visual illusions (Tversky and Kahneman, 1974), and strong *framing* effects on decisions (Tversky and Kahneman, 1981).

With regard to Bayesian problems, the rationality of human reasoning is routinely discussed in terms of *inverting* or *revising* beliefs (e.g., Baratgin and Politzer, 2006; Koehler, 1996). In their accounts of the *Monty Hall problem*, Krauss and Wang (2003) employ the notion of *perspective change* in both a literal and figurative sense, and Baratgin (2009) distinguishes the experimenters' *focusing* situation from participants' *updating* interpretation.

Similarly, the 2×2 matrix construct features prominently in textbooks on research methodology and statistics. Falk (1986) suggests using a two-dimensional *frequency table* as a "didactic device" and mentions the benefit that "the two orthogonal directions for the computation of the two inverse conditional probabilities will be conspicuous" (p. 296). Fiedler et al. (2000) use binary contingency tables to study the interplay of numeric formats and sampling processes on alleged biases in judging conditional probabilities.

As this list of influences could be continued, our contribution lies mainly in combining some of them, anchoring them in an analysis of a shared representational construct, and suggesting the notion of adopting perspectives as an explanatory mechanisms for phenomena that are typically discussed in terms of Bayesian updating or representational formats. Our approach reveals structural similarities between problems and theoretical discussions, and links a far-flung array of concepts, measures, and domains.

Given that the structural notion of a 2×2 matrix is the core construct of our model, we should address a question regarding its ontological status: Should we think of the 2×2 matrix as an external or as an internal representation? The answer is, perhaps predictably, a matter of perspective: We mostly view the 2×2 matrix as an external representation that helps us analyze and explicate a set of tasks and problems that are based on frequency counts, binary contingencies, and conditional probabilities. For instance, our visualizations (see Figures 3–11) and icons like 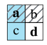 (see Table 3) provide external representations that allow illustrating how specific tasks are performed. These tasks can be distinguished by adopting particular perspectives on a shared 2×2 matrix construct. The 2×2 matrix is well-suited for our present purposes, as it provides an abstract, yet structurally complete representation. Despite its compact form, it preserves the three-dimensional nature of the underlying task structure, as opposed to its informationally equivalent alternatives. Crucially, the key 2×2 matrix promoted in this paper is not an external visualization, but a theoretical *construct* that merely can be visualized as a two-dimensional diagram or table. This visualization is a compact 2D-projection of the 3D-semantics that faithfully represent the structure of tasks in various task domains. Although a 2×2 matrix also imposes representational constraints (e.g., two explicit orthogonal dimensions vs. one implicit diagonal dimension), these constraints accurately reflect the semantic structure of many task domains (in the case of the 2×2 matrix $\{X, Y\}$, in which the diagonal implicitly represents the correspondence dimension Z). All alternative 2D-representations either introduce additional representational artifacts (e.g., lists, trees, and unit squares impose an order of dimensions) or explicate information that is fully contained in the 2×2 matrix (e.g., natural frequencies, frequency nets).

The boundaries between external and internal representations are often blurred. When a particular way of dissecting and organizing a domain appears to fit extremely well to the underlying structure of the problems, both external and internal representations can be turned into methodological tools. If an exploration based on some structural construct is successful — in the sense of providing insights and showing links and directions that we have not recognized before — it can happen that external representations are internalized and subsequently guide and shape our thoughts. Thus, the construct of a 2×2 matrix — due to its chameleon-like ability to capture and reflect a large variety of measures and tasks — is both an external and internal representation of tremendous potential. As thinking in terms of 2×2 matrices has been a clarifying and productive endeavor for us, we trust that readers will discover opportunities for framing additional problems in this form.

REFERENCES

Baratgin, J. (2009). Updating our beliefs about inconsistency: The Monty-Hall case. *Mathematical Social Sciences* 57, 67–95. doi:10.1016/j.mathsocsci.2008.08.006

Baratgin, J. and Politzer, G. (2006). Is the mind Bayesian? The case for agnosticism. *Mind & Society* 5, 1–38. doi:10.1007/s11299-006-0007-1

Binder, K., Krauss, S., and Wiesner, P. (2020). A new visualization for probabilistic situations containing two binary events: The frequency net. *Frontiers in Psychology* 11, 750. doi:10.3389/fpsyg.2020.00750

Bortz, J. and Schuster, C. (2010). *Statistik für Human- und Sozialwissenschaftler* (Berlin; Heidelberg; New York: Springer-Verlag)

Brunswik, E. (1952). *The conceptual framework of psychology* (Chicago, IL: University of Chicago Press)

De Finetti, B. (1937). La prévision: Ses lois logiques, ses sources subjectives. *Annales de l'institut Henri Poincaré* 7, 1–68

Falk, R. (1986). Conditional probabilities: Insights and difficulties. In *Proceedings of the Second International Conference on Teaching Statistics*. 292–297

Fiedler, K., Brinkmann, B., Betsch, T., and Wild, B. (2000). A sampling approach to biases in conditional probability judgments: Beyond base rate neglect and statistical format. *Journal of Experimental Psychology: General* 129, 399–418. doi:10.1037/0096-3445.129.3.399

Hammond, K. R. and Stewart, T. R. (2001). *The essential Brunswik: Beginnings, explications, applications* (New York, NY: Oxford University Press)

Howell, D. C. (2013). *Statistical Methods for Psychology* (Wadsworth, Cengage Learning, Belmont, CA), 8th International edn.

Kempf, W. (2006). *Forschungsmethoden der Psychologie. Zwischen naturwissenschaftlichem Experiment und sozialwissenschaftlicher Hermeneutik. Band 1: Theorie und Empirie* (Berlin: Regener)

Koehler, J. J. (1996). The base rate fallacy reconsidered: Descriptive, normative, and methodological challenges. *Behavioral and Brain Sciences* 19, 1–17. doi:10.1017/S0140525X00041157

Köhler, W. (1925). *The mentality of apes* (New York, NY: Harcourt Brace Jovanovich)

Krauss, S. and Wang, X.-T. (2003). The psychology of the Monty Hall problem: Discovering psychological mechanisms for solving a tenacious brain teaser. *Journal of Experimental Psychology: General* 132, 3–22. doi:10.1037/0096-3445.132.1.3

Kurz-Milcke, E., Gigerenzer, G., and Martignon, L. (2008). Transparency in risk communication: Graphical and analog tools. *Annals of the New York Academy of Sciences* , 18–28doi:10.1196/annals.1399.004

Larkin, J. H. and Simon, H. A. (1987). Why a diagram is (sometimes) worth ten thousand words. *Cognitive Science* 11, 65–100. doi:10.1111/j.1551-6708.1987.tb00863.x

Leonhart, R. and Lichtenberg, S. (2009). *Lehrbuch Statistik. {E}instieg und Vertiefung* (Bern: Hogrefe), 2 edn.

Shepard, R. N. (2001). Perceptual-cognitive universals as reflections of the world. *Behavioral and Brain Sciences* 24, 581–601. doi:10.1017/S0140525X01000012

[Dataset] Statistical hypothesis testing (2020). Statistical hypothesis testing — Wikipedia, the free encyclopedia. [online; accessed 25-Nov-2020]

Tversky, A. and Kahneman, D. (1974). Judgment under uncertainty: Heuristics and biases. *Science* 185, 1124–1131. doi:10.1126/science.185.4157.1124

Tversky, A. and Kahneman, D. (1981). The framing of decisions and the psychology of choice. *Science* 211, 453–458. doi:10.1126/science.7455683

Wassner, C., Martignon, L., and Biehler, R. (2004). Bayesianisches Denken in der Schule (Bayesian inference in school). *Unterrichtswissenschaft* 32, 58–96

Wertheimer, M. (1959). *Productive thinking* (New York, NY: Harper & Row)
